# Statistical Estimation of Effects of Implemented Government Policies on COVID-19 Situation in South Korea

**DOI:** 10.3390/ijerph18042144

**Published:** 2021-02-22

**Authors:** Gyujin Heo, Catherine Apio, Kyulhee Han, Taewan Goo, Hye Won Chung, Taehyun Kim, Hakyong Kim, Yeonghyeon Ko, Doeun Lee, Jisun Lim, Taesung Park

**Affiliations:** 1 Interdisciplinary Program in Bioinformatics, Seoul National University, Seoul 08826, Korea; hgj0106@snu.ac.kr (G.H.); 2019-20240@snu.ac.kr (C.A.); hgh1031@snu.ac.kr (K.H.); gootec92@snu.ac.kr (T.G.); 2corin417@snu.ac.kr (D.L.); 2 Department of Chemistry, Seoul National University, Seoul 08826, Korea; hyewon.chung@snu.ac.kr; 3 Department of Statistics, Seoul National University, Seoul 08826, Korea; qeeqee10@snu.ac.kr (T.K.); newstellar@snu.ac.kr (Y.K.); 4 Department of Industrial Engineering, Seoul National University, Seoul 08826, Korea; gkrdyd111@snu.ac.kr; 5 Department of Archeology and Art History, Seoul National University, Seoul 08826, Korea; 6 The Research Institute of Basic Sciences, Seoul National University, Seoul 08826, Korea; swanjslim@gmail.com

**Keywords:** SARS-COV-2, COVID-19, pandemic, policies, indices, stringency index, lagging, segmented Poisson model

## Abstract

Since the outbreak of novel SARS-COV-2, each country has implemented diverse policies to mitigate and suppress the spread of the virus. However, no systematic evaluation of these policies in their alleviation of the pandemic has been done. We investigate the impact of five indices derived from 12 policies in the Oxford COVID-19 Government Response Tracker dataset and the Korean government’s index, which is the social distancing level implemented by the Korean government in response to the changing pandemic situation. We employed segmented Poisson model for this analysis. In conclusion, health and the Korean government indices are most consistently effective (with negative coefficients), while the restriction and stringency indexes are mainly effective with lagging (1~10 days), as intuitively daily confirmed cases of a given day is affected by the policies implemented days before, which shows that a period of time is required before the impact of some policies can be observed. The health index demonstrates the importance of public information campaign, testing policy and contact tracing, while the government index shows the importance of social distancing guidelines in mitigating the spread of the virus. These results imply the important roles of these polices in mitigation of the spread of COVID-19 disease.

## 1. Introduction

The first confirmed case of severe acute respiratory syndrome coronavirus 2 (SARS-CoV-2) infection in South Korea was imported from China on 20 January 2020, followed by the detection of one or two cases on average in the subsequent days. By 10 February2020, there were 28 cases of laboratory-confirmed COVID-19 in South Korea [1,2]. However, the number of confirmed cases of SARS-CoV-2 infection started to increase rapidly on 19 February 2020, with a total of 31,004 confirmed COVID-19 cases and 509 deaths reported as of 23 November 2020, according to the Korea Centers for Disease Control and Prevention [3].

The epicenter of South Korean COVID-19 outbreak has been identified in Daegu, a city of 2.5 million people, approximately 150 miles South East of Seoul. The rapid spread of COVID-19 in South Korea has been attributed to one case linked to a super spreading event that led to more than 5213 secondary cases stemming from church services in the city of Daegu [1,2]. From 6 May 2020, several COVID-19 cases were also confirmed among persons who had visited nightclubs in Itaewon (an area surrounding Itaewon-dong known as Seoul’s most diverse and foreigner-friendly district, proximity to Yongsan Garrison US Military Base and for nightlife, tourist attractions) [4], during the 30 April–5 May holiday. Secondary transmission by case-patients linked to the Itaewon nightclubs led to at least 246 local transmission of COVID-19 in other parts of the country [5]. These few clusters and others have become the primary driving force of the infection in South Korea [1,2].

Emerging and re-emerging infectious diseases are undoubtedly one of humankind’s most important health and security risks. Since threats from known or new epidemic outbreaks increases so is the potential impact of mathematical models, statistical inferences and simulation approaches in guiding prevention and mitigation plans [6]. As the recent 2013–2016 Ebola epidemic exemplified and now the ongoing COVID-19 pandemic, an infectious disease outbreak often forces public health officials and governments to make key decisions to mitigate the outbreak in a changing environment where multiple factors positively or negatively impact local disease transmission [7]. In general, governments can take two distinctive strategies: mitigation and suppression. The former aims at lowering maximum healthcare demand by reducing contagion rates through non-pharmaceutical interventions, while the latter approach adopts very restrictive measures to push down the prevalence of new cases to zero [8,9].

The COVID-19 pandemic has forced societies and governments around the world to respond with unprecedented policies such as closing schools and restricting populations to their homes, designed to slow the growth rate of infections, as inferred from past epidemics [10]. However, the actual effects of these policies on infection rates in the ongoing pandemic are unknown. Societies around the world are considering whether the health benefits of these anti-contagion policies are worth their social and economic costs. Many of these costs are clearly observed; for example, business restrictions increase unemployment and school closures affect educational outcomes, which also elicites resistance from these affected groups of people [10,11].

South Korea’s response is considered by many as one the most effective models against COVID-19. South Korea flattened the curve of COVID-19 before, whereby the average number of new cases per day fell to 6.4 in the first week of May, by combining testing, early isolation, and the free treatment of positive cases combined with digital technologies without taking to “lockdown” measures [12]. However, the South Korean approach to COVID-19 of; testing and early isolation, two days per week transparent press briefings on COVID-19, use of information technology and voluntary engagement of citizens and businesses, may be difficult to emulate even for countries like USA and the UK, [13]. In particular, the current worsening of COVID-19 situation in the country mainly in the metropolitan areas, is tied to small church gatherings, restaurants and schools nationwide [14,15,16].

Our objective is to determine whether the guidelines implemented so far in South Korea have been effective in the mitigation and suppression of the spread of COVID-19 in the country. Here, Closing, Restriction, Economic and Health indices, calculated as arithmetic means from the Oxford COVID-19 Government Response Tracker (OxCGRT) dataset of Blavatnik School of Government and the University of Oxford [17], contains 17 policies implemented by governments around the globe (Supplementary Table S1). These policies are recorded in numeric and ordinal scales, a measure of the strength (levels) of the policy in addition to the stringency index, which demonstrates the overall measure of strictness of the government responses and the Korean Government index; the social distancing levels enforced by the Korean government in response to the pandemic situation in the country. These indices are analyzed against COVID-19 daily confirmed cases obtained from Kaggle [18] and the public portal data provided by the Korea’s Ministry of Health and Welfare [19].

## 2. Materials and Methods

### 2.1. COVID-19 Confirmed Cases Data

The daily series of confirmed cases of COVID-19 for South Korea from 20 January 2020 to 20 November 2020 was obtained from Kaggle (from 20 January to 30 June) [18] and Korea public data portal of the Ministry of Health and Welfare (from 1 July to 20 November) [19]. The combined data was divided into two regions: Seoul Metropolitan area (SeoulMetro; Seoul, Incheon and Gyeonggi-do) and non-Seoul Metropolitan area (non-SeoulMetro; other cities beside Seoul, Incheon, and Gyeonggi-do). The analysis was conducted on the Domestic area (SeoulMetro and Non-SeoulMetro), Seoul Metropolitan area, and non-Seoul Metropolitan area data, respectively. The data was split into training (20 January~10 November) and test data (the last 10 days, 11 November to ~20 November) for downstream analysis with the test data used for prediction analysis.

### 2.2. Oxford COVID-19 Government Response Tracker (OxCGRT) Data

Data on government policies implemented to combat the COVID19 spread was retrieved from Blavatnik School of Government and the University of Oxford, The Oxford COVID-19 Government Response Tracker (OxCGRT) dataset [17]. OxCGRT systematically collects information on several different common policy responses that governments have taken to respond to the pandemic on 17 indicators, from more than 160 countries (Table S1). Eight of the policy indicators (C1-C8) record information on containment and closure policies, such as school closures and restrictions in movement. Four of the indicators (E1–E4) record economic policies, such as income support to citizens or provision of foreign aid. Five of the indicators (H1–H5) record health system policies such as the COVID-19 testing regime or emergency investments into healthcare [19].

**Figure 1:**
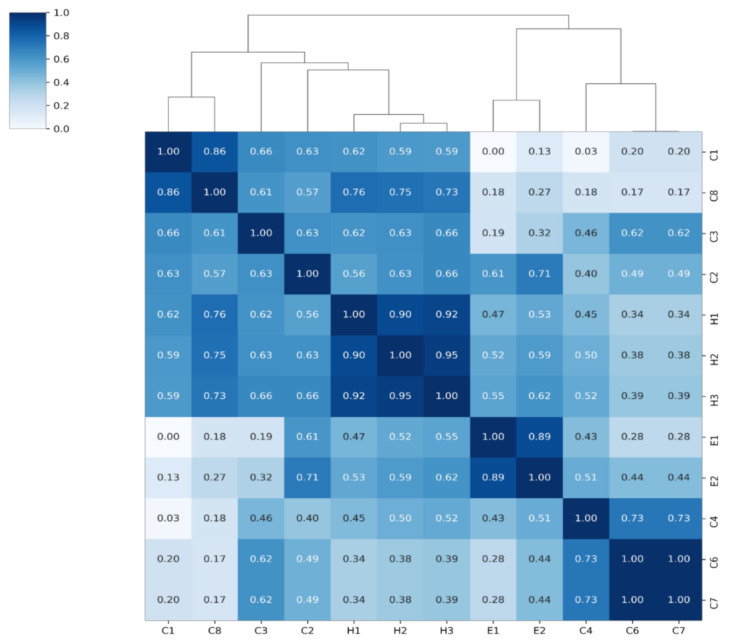
Heat map of the clustered Policies. The Pearson’s correlation matrix of the clustered polices using the hierarchical clustering with 1-correlation distance. H1, H2, H3 form the Health Index, E1, E2 form the Economic Index, C4, C6 and C7 form the Restriction Index while C1, C8, C2 and C3 form the Closing Index.

**Figure 2:**
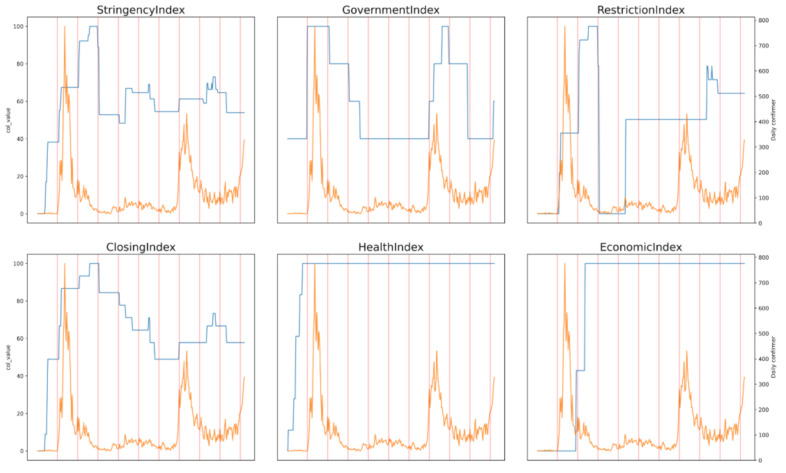
Variation of Indices with Daily Confirmed Cases Over Time (days). The blue line indicates the index’s level and orange line indicates the COVID-19 daily confirmed cases of South Korea. Each vertical red line represents 30 days difference at x-axis, approximately a month.

Of the 17 government implemented policies (Table S1), 12 are aggregated into a set of four indices (Table 1) determined by hierarchical clustering analysis with 1-Pearson’s correlation distance as shown in Figure 1. The policies with the strongest correlation between them and from the same category (starting with C, H or E) were used to calculate indices as shown in the Supplementary Equations S1 and S2. The indices are calculated by averaging the values of clustered policies. Stringency index is provided by OxCGRT which shows overall measure of strictness of government response while the Korean government index quantified from the Korean government’s social distancing levels implemented during the course of the pandemic by the Korean government up to 17 November 2020, which is different from that of the OxCGRT dataset (Table 2) [20]. The variation of the calculated indices with COVID-19 daily confirmed cases is shown in Figure 2. However, C5 variable was excluded from the research because it was not applied in South Korea.

### 2.3. Segmented Poisson Model

In this study, we simply regarded the daily new cases and the policy indices as a function of time t based on a segmented Poisson model. Let $Y_{t}$ be the daily new cases at day *t* which is the number of days since first case occurred. Poisson model is defined as

(1) $$Y_{t}\sim \mathrm{Poisson}\left(\mu_{t}\right),$$

where $\mu_{t}$ is the expectation of $Y_{t}$ with segments.

**Figure 3:**
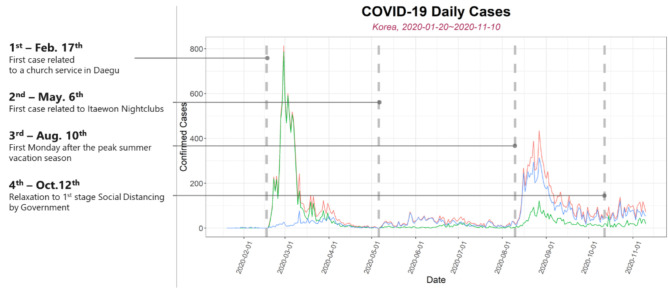
Daily Confirmed Cases of South Korea with the Breakpoint Information. Daily confirmed cases of Domestic, SeoulMetro, Non-SeoulMetro is represented in red, blue, and green respectively.

Breakpoints were considered in the daily confirmed cases (Figure 3) during the analysis by splitting the daily confirmed cases into segments: (i) 17 February—the first case related to a church service in Daegu, (ii) 6 May—the first case related to Itaewon nightclub, (iii) 10 August, the first Monday after the peak summer vacation season and (iv) 20 October relaxation to first stage social distancing in South Korea. The segments are introduced to capture information in the peaks and flattened areas of the curve during the analysis. These breakpoints were decided using some of the aforementioned significant events linked to the spread of COVID-19 in South Korea.

Lagging (*l*), from 0 to 10 days, was also introduced since intuitively, the policies of day (*t*) will affect confirmed cases after few days (*t + l*) not the day the policy was applied. In other words, daily confirmed case of day (*t*) is determined by policies of day (*t − l*). The Poisson model is fitted without, with single (an index at a time) and multiple indices (all policies at once). 

Since there are four breakpoints, five segments are defined as follows:

(2) $$\log\left(\mu_{t}\right)\;=\;\left\{\begin{array}{cc} \beta_{0}\;+\;\beta_{11}t\;+\;\beta_{21}\log\left(t\;+\;1\right)\;+\;\beta_{p}Index_{t-l},\;|\; & \left(t=0,1,\ldots ,c_{1}-1\right)\\ \beta_{0}\;+\;\beta_{11}t\;+\;\beta_{21}\log\left(t\;+\;1\right)\;+\;\beta_{12}\left(t-c\right)\;+\;\beta_{22}\log\left(t-c\;+\;1\right)\;+\;\beta_{p}Index_{t-l},\;|\; & \left(t=c_{1},\ldots ,c_{2}-1\right)\\ \beta_{0}\;+\;\beta_{11}t\;+\;\beta_{21}\log\left(t\;+\;1\right)\;+\;\ldots \;+\;\beta_{13}\left(t-c\right)\;+\;\beta_{23}\log\left(t-c\;+\;1\right)\;+\;\beta_{p}Index_{t-l},\;|\; & \left(t=c_{2},\ldots ,c_{3}-1\right)\\ \beta_{0}\;+\;\beta_{11}t\;+\;\beta_{21}\log\left(t\;+\;1\right)\;+\;\ldots \;+\;\beta_{14}\left(t-c\right)\;+\;\beta_{24}\log\left(t-c\;+\;1\right)\;+\;\beta_{p}Index_{t-l},\;|\; & \left(t=c_{3},\ldots ,c_{4}-1\right)\\ \beta_{0}\;+\;\beta_{11}t\;+\;\beta_{21}\log\left(t\;+\;1\right)\;+\;\ldots \;+\;\beta_{15}\left(t-c\right)\;+\;\beta_{25}\log\left(t-c\;+\;1\right)\;+\;\beta_{p}Index_{t-l},\;|\; & \left(t=c_{4},\ldots ,n\right) \end{array}\right.$$

where $c_{i}\left(i=0,1,2,3,4\right)$are breakpoints.

To evaluate single index models and multiple indices model to the model without indices, mean squared errors (MSEs) for the train and test datasets for each of the fitted models were calculated as follows:

(3) $$MSE=\frac{1}{n}\sum_{t=1,\ldots ,n}{\left(y_{t}-\hat{\mu_{t}}\;\right)}^{2},$$

where *n* is the number of data points, $y_{t}$ is the observed values, $\hat{\mu_{t}}$ is the predicted values from a fitted model. The mean squared error is used as a default metric for evaluation of most regression models. All analysis was done with R version 3.6.3.

## 3. Results

To adjust for the differences between indices, each policy was divided by possible maximum level of each data and multiplied by 100, thus, in a scale of 0 to 100, as shown in equations S1 and S2 of the Supplementary Materials. The result interpretations mainly centered around the coefficients of the indices in that for one-unit change in the index, the difference in the logs of expected counts (COVID-19 daily confirmed cases) is expected to change by the respective regression coefficient, given the other indices in the model are held constant. Therefore, an index with a negative coefficient (β_p_) is effective as it has an impact in lowering the COVID-19 daily confirmed cases while those with a positive coefficient lead to the increase in the number of COVID-19 daily confirmed cases, i.e., no impact in this case.

The analysis was divided into Seoul Metropolitan area (SeoulMetro), Non-SeoulMetro and domestic (SeoulMetro + Non-SeoulMetro) areas. For SeoulMetro: from both single index and multiple indices models, the coefficients decrease with lagging with stringency index having the largest coefficients followed by economic index and then restriction index in single index models while closing index has the largest coefficients in multiple indices model. The Korean government and health indices (Table 3) are the only indices with negative coefficients. This demonstrates the impact of the respective policies in lowering the number of COVID-19 daily confirmed cases, hence, mitigation and suppression of the pandemic. In the multiple indices model, health index and Korean government indices are the two indices with negative coefficients when lagging is considered (Table 4). For non-SeoulMetro; economic index followed by stringency index has the largest coefficients for single index models while health index, economic index and Korean government index have the largest coefficients for multiple indices model. Also, restriction and stringency indices are effective at 10-days’ lag (−0.0001, −0.0197 respectively), closing index after 8-days’ lag (−0.0109, −0.0188) and health index only from 0-day to 5-days’ lag (−0.6493, −0.4707, −0.3280, −0.2244, −0.1469, −0.0641) while Korean government, economic and stringency indices coefficients all remain positive for single index model (Table 5). For multiple indices model: closing index is effective after 5-days’ lag (−0.0007, −0.0025, −0.0015, −0.0194, −0.0257) and health index from 0~2-days’ lag only (−0.7129, −0.3738, −0.0785) (Table 6). At country level (domestic); health index followed by economic index has the largest coefficients for both single index model and multiple indices model. Also, health index (−0.1727, −0.1170, −0.0765, −0.0397, −0.0014; 0~4-days’ lag), closing index (−0.0046, −0.0216, −0.0304; 8~10-days’ lag), Korean government and stringency indices (−0.0001, −0.0040 respectively; at 10-days’ lag) were effective (Table 7). For multiple indices model (Table 8): closing index (−0.0030, −0.0092, −0.0239, −0.0287; 7~10-days’ lag) and health index (−0.1015, −0.0367; 0~1-days’ lag) were effective.

Next, we compare MSE for the model without indices to models with indices. In the case of SeoulMetro: test MSE is higher than train MSE, both single index models and multiple indices model generally have lower train MSEs than that of the model with no indices (937.7) irrespective of lagging, except for health index and similar range test MSE (6930.6) and test MSE increases with lagging. However, the test MSE for stringency index is far lower than that of no policy model (Table S3). Secondly, for non-SeoulMetro, no policy model on average have lower test MSE (881.5) than that of the single index models and multiple indices model but higher train MSE (1509.6). Test MSE generally decreases with lagging for stringency index, restriction index, Korean government index and multiple indices model while it increases for closing index, economic index and health index but stringency index still has a lower test MSE than no policy model MSE (Table S6). Lastly, for Domestic: train MSE is lower than test MSE. Models with indices generally have lower train MSEs than the no policy model (4340.1) while test MSE increases with lagging but similar in range with that of no policy model (13,809.1) (Table S9).

We also performed projection analysis of the trends of confirmed cases under the absence of any index and at different levels of indices which in turn captures the trend of confirmed cases at different policies’ levels. For SeoulMetro, prediction shows that the daily confirmed cases for South Korea rises for some time but closing index (Figure S2), health index (Figure S3) and Korean government index (Figure S4) estimate the lowest number of COVID-19 daily confirmed cases at the most stringent level of indices and the impact of these indices increases with lagging since time is required before their effect come into play. This demonstrates the importance of contact tracing and testing policy from the onset of the disease. For non-SeoulMetro with, or without, an index in place and irrespective of lagging, we see a rapid continuous rise of COVID-19 cases in a very short period of time, however, we still see the impact of these indices especially closing (Figure S6), restriction (Figure S7), stringency (Figure S8) indices with time (lagging), in lowering the spread of the virus compared to when no policy is put in place (Figure S5). The whole country prediction trends (domestic) display the same patterns as those for SeoulMetro (Figures S9–S13).

Closing index, Korean government index and especially health index always produce negative coefficients with or without lagging. Prediction plots of trends of COVID-19 daily confirmed cases under different index levels also demonstrates these three as being the most effective indices in lowering the number of cases to zero in a short time.

## 4. Discussion

The objective of our analysis was to determine whether the government policies implemented so far have impacted the pandemic in reducing the daily number of COVID-19 confirmed cases. The effectiveness of health index with or without lagging, demonstrates the importance of public information campaign that equips the general public with information about the etiology of the disease, transmission paths and management, the testing and contact tracing policies that have been paramount in South Korea for control of the spread of the pandemic. The closing index, which encompasses school closure, workplace closure, cancel of public events and international travel restriction actions, is only effective with lagging, which demonstrates that time is needed before the impact of the policies can be observed. Also, this may be due to a reduction in the number of imported cases from other countries, and the prevention of conditions or environments that create a super spreading event like close proximity at workplaces or schools, which these policies target. The restriction index has a negative coefficient with lagging, thus restriction on gathering, stay at home requirements, and domestic travel restriction policies aim to avoid mixing of the people in public so as to slow down the spread of the infection.

The general lack of effective results (negative coefficients) with the stringency index mainly with the prediction trends of COVID-19 cases especially in Non-Seoul metropolitan areas, shows low level of seriousness from government or concerned agencies in making individuals comply with the policies being implemented which demonstrates a call for government to tighten the strictness of observing these policies by the public. Daegu, the epicenter of the first wave is located in this region, which demonstrates restrictions on some gatherings, like churches maybe should be elevated.

South Korea’s rapid adoption of the “test, trace, isolate, and treat” strategy where individuals with suspected disease were tested, contacts identified, strict isolation enforced, and free treatment given to those infected [13], with compensation for people who had to self-isolate is clearly seen, especially between 1 March to 1 August (Figure 3) and this provides important policy implications for other countries and shows the needs for strengthening three core competencies: Digital technology, efficient health governance, and civic partnership [12].

“This was a population-based study that mainly focused on the impact of policies adopted on COVID-19 cases in Korea. One cannot ignore the evidence that patients’ clinical features (age, pre-existing comorbidities, etc.) affect the severity of COVID-19 infection as they are more prone to developing COVID-19 severe symptomatic conditions [21,22,23,24]. However, our current analysis focuses only on the population level information, which makes it difficult to take into account individual level clinical features directly. Therefore, in future studies, it would be desirable to develop an aggregated model, which can consider these individual clinical features. Such models are expected to reduce bias due to underestimation of healthy asymptotic COVID-19 people not confirmed by SARS-COV-2 antibody tests [25,26].

## 5. Conclusions

Korean government index, health index and closing index are the most consistently effective indices among all the indices with or without lagging. The effectiveness of these indices shows that with the ongoing rise in the number of COVID-19 daily confirmed cases in the country, a strengthening of the government index, health policies under the health index, and closure of hotspots that may related to super spreading events can easily flatten the curve again as in it did in May 2020. Lagging also demonstrated that some time is required, even up to ten days for some policies, before their impact in lowering the number of cases can be observed.

## Figures and Tables

**Table 1:** Polices used for the calculation of the Indices. The Korean government index represents the Korean government’s social distancing levels implemented during the course of the pandemic by the Korean government.

Index Name	Policies Used in Index from OxCGRT Dataset	Simple Explanation
Restriction Index (RI)	C4: Restriction on gatherings C6: Stay at home requirements C7: Domestic travel restrictions	Consists of restrictions on meeting, restrictions on going out and domestic travel. The average value of policies related to the movement and contact of people.
Closing Index (CI)	C1: School closing C2: Workplace closing C3: Cancel public events C8: International travel controls	Consists of suspension of school, telecommuting, cancellation of public events and travel regulations abroad. The average value of a variable that regulates the gathering of many public events, companies, etc.
Economic Index (EI)	E1: Income support E2: Debt/contract relief	The average value of the government’s financial support policy indices.
Health Index (HI)	H1: Public information campaign H2: Testing policy H3: Contact tracing	The average value of the government’s health care policy indices.

**Table 2:** Korean government Index. The Korean Government Index is quantified from the Korean government’s social distancing levels issued by the Korean government in response to the COVID-19 situations in the country.

Levels	Idea	Level Description
1.0	Distancing in life	Mandatory quarantine rules for high-risk activities and facilities
1.5	Workplace closing	Epidemic region: Strengthen quarantine measures, such as limiting the number of multi-use facilities, in order to block the trend through dangerous facilities and activities Other region: Maintain the first stage, but take self-governing measures according to the quarantine situation, such as the possibility of radio waves.
2.0	Cancel public events	Epidemic region: Prohibit gatherings and events of more than 100 people, bans gatherings of entertainment facilities, allows only packaging and delivery of restaurants after 21:00, expands restrictions on the use of facilities, and mandates the wearing of indoor masks. Other region: The principle of implementing the key measures in step 1.5, and autonomous measures by local governments according to the quarantine situation.
2.5	Restriction on gathering	Ban gatherings and events of more than 50 people nationwide, banning gatherings such as singing practice sites, suspending major multi-use facilities after 21:00.
3.0	Closing public transport	Banning gatherings and events of more than 10 people nationwide, and discontinuing the operation of all multi-use facilities other than essential facilities;

**Table 3:** Seoul Metropolitan Area’s Coefficients from Single Index Model.

Lag	Closing Index	Restriction Index	Economic Index	Health Index	Stringency Index	Korean Government Index
No lagging	0.0688	0.0118	0.0354	−0.0247	0.0623	0.0137
1	0.0609	0.0115	0.0356	0.0294	0.0558	0.0100
2	0.0605	0.0124	0.0368	0.0284	0.0579	0.0084
3	0.0501	0.0152	0.0332	0.0524	0.0587	0.0058
4	0.0393	0.0174	0.0327	0.1177	0.0583	0.0033
5	0.0328	0.0156	0.0303	−0.0550	0.0542	−0.0016
6	0.0197	0.0145	0.0262	−0.1056	0.0457	−0.0055
7	0.0098	0.0148	0.0265	−0.0825	0.0414	−0.0090
8	−0.0025	0.0133	0.0317	−0.0631	0.0317	−0.0147
9	−0.0046	0.0122	0.0344	−0.0771	0.0266	−0.0179
10	−0.0090	0.0100	0.0357	−0.0652	0.0192	−0.0197

**Table 4:** Seoul Metropolitan Area’s Coefficients from Multiple Indices Model.

Lag	Closing Index	Restriction Index	Economic Index	Health Index	Korean Government Index
No lagging	0.0625	0.0086	0.0286	−0.0745	0.0159
1	0.0541	0.0080	0.0292	−0.0286	0.0113
2	0.0532	0.0089	0.0306	−0.0620	0.0096
3	0.0411	0.0131	0.0247	−0.0902	0.0080
4	0.0301	0.0155	0.0225	−0.0416	0.0058
5	0.0268	0.0124	0.0246	−0.1015	−0.0001
6	0.0166	0.0110	0.0231	−0.1193	−0.0043
7	0.0090	0.0112	0.0237	−0.0959	−0.0077
8	0.0022	0.0089	0.0306	−0.0818	−0.0137
9	0.0040	0.0075	0.0355	−0.0963	−0.0175
10	0.0022	0.0061	0.0367	−0.0854	−0.0194

**Table 5:** Non-Seoul Metropolitan Area’s Coefficients from Single Index Model.

Lag	Closing Index	Restriction Index	Economic Index	Health Index	Stringency Index	Korean Government Index
No lagging	0.0576	0.0167	0.0408	−0.6493	0.0546	0.0263
1	0.0552	0.0177	0.0533	−0.4707	0.0558	0.0249
2	0.0573	0.0181	0.0557	−0.3280	0.0573	0.0239
3	0.0573	0.0246	0.0534	−0.2244	0.0680	0.0217
4	0.0462	0.0288	0.0598	−0.1469	0.0706	0.0213
5	0.0408	0.0311	0.0578	−0.0641	0.0738	0.0116
6	0.0243	0.0296	0.0597	0.0072	0.0637	0.0112
7	0.0136	0.0197	0.0683	0.0629	0.0453	0.0110
8	0.0063	0.0127	0.0751	0.1137	0.0303	0.0160
9	−0.0109	0.0054	0.0820	0.1091	0.0093	0.0166
10	−0.0188	−0.0001	0.0841	0.0844	−0.0023	0.0115

**Table 6:** Non-Seoul Metropolitan Area’s Coefficients from Multiple Indices Model.

Lag	Closing Index	Restriction Index	Economic Index	Health Index	Korean Government Index
No lagging	0.0297	0.0121	0.0276	−0.7129	0.0349
1	0.0357	0.0072	0.0384	−0.3738	0.0299
2	0.0518	0.0015	0.0381	−0.0785	0.0281
3	0.0483	0.0095	0.0286	0.0481	0.0304
4	0.0308	0.0183	0.0303	0.0756	0.0307
5	0.0211	0.0222	0.0288	0.1042	0.0190
6	−0.0007	0.0287	0.0302	0.1185	0.0160
7	−0.0025	0.0186	0.0491	0.1338	0.0130
8	−0.0015	0.0132	0.0572	0.1895	0.0192
9	−0.0194	0.0155	0.0610	0.1610	0.0189
10	−0.0257	0.0132	0.0649	0.1116	0.0133

**Table 7:** Domestic Coefficients from Single Index Model.

Lag	Closing Index	Restriction Index	Economic Index	Health Index	Stringency Index	Korean Government Index
No lagging	0.0770	0.0178	0.0404	−0.1727	0.0651	0.0188
1	0.0707	0.0196	0.0510	−0.1170	0.0664	0.0174
2	0.0688	0.0210	0.0561	−0.0765	0.0695	0.0179
3	0.0634	0.0257	0.0568	−0.0397	0.0758	0.0174
4	0.0502	0.0288	0.0630	−0.0014	0.0767	0.0170
5	0.0410	0.0290	0.0637	0.0277	0.0757	0.0114
6	0.0219	0.0270	0.0657	0.0546	0.0642	0.0092
7	0.0073	0.0205	0.0722	0.0865	0.0476	0.0069
8	−0.0046	0.0144	0.0793	0.1179	0.0304	0.0062
9	−0.0216	0.0079	0.0851	0.1236	0.0101	0.0044
10	−0.0304	0.0020	0.0878	0.1211	−0.0040	−0.0001

**Table 8:** Domestic Coefficients from Multiple Indices Model.

Lag	Closing Index	Restriction Index	Economic Index	Health Index	Korean Government Index
No lagging	0.0654	0.0071	0.0256	−0.1015	0.0215
1	0.0582	0.0069	0.0355	−0.0367	0.0183
2	0.0572	0.0069	0.0390	0.0149	0.0179
3	0.0483	0.0137	0.0337	0.0521	0.0184
4	0.0327	0.0194	0.0362	0.0716	0.0186
5	0.0253	0.0201	0.0392	0.0828	0.0123
6	0.0058	0.0226	0.0418	0.0774	0.0102
7	−0.0030	0.0182	0.0526	0.0859	0.0073
8	−0.0092	0.0149	0.0601	0.1057	0.0069
9	−0.0239	0.0142	0.0637	0.0891	0.0056
10	−0.0287	0.0115	0.0655	0.0749	0.0020

## Data Availability

Publicly available datasets were analyzed in this study. These datasets can be found at the data links provided in the references.

## Supplementary Materials

The following are available online at https://www.mdpi.com/1660-4601/18/4/2144/s1, Figure S1: Predicted confirmed cases without any policy implemented, Figure S2: Trends of confirmed cases at different levels of Closing Index with 10-days’ lag, Figure S3: Trends of confirmed cases at different levels of Health Index with 6-days’ lag, Figure S4: Trends of confirmed cases at different levels of Korean Government Index with 10-days’ lag, Figure S5: Trend of confirmed cases with no policies implemented, Figure S6: Trends of confirmed cases at different levels of Closing Index with 10-days’ lag, Figure S7: Trends of confirmed cases at different levels of Restriction Index with 10-days’ lag, Figure S8: Trends of confirmed cases at different levels of Stringency Index with 10-days’ lag, Figure S9: Trends of confirmed cases with no policies implemented, Figure S10: Trends of confirmed cases at different levels of Closing Index, Figure S11: Trends of confirmed cases at different levels of Health Index, Figure S12: Trends of confirmed cases at different levels of Stringency Index, Figure S13: Trends of confirmed cases at different levels of Government Index, Table S1: Summary of Policy Variables Used for Analysis. The 13 government implemented policies recorded on an ordinal scale that represents the strength of the policy, Table S2: Seoul Metropolitan Area Coefficients from Single Index Model, Table S3: Seoul Metropolitan Area Coefficients from Multiple Indices Model, Table S4: Seoul Metropolitan Area MSE Summary for Single and Multiple Indices Models, Table S5: Non-Seoul Metropolitan Area Coefficients from Single Index Model, Table S6: Non-Seoul Metropolitan Area Coefficients from Multiple Indices Model, Table S7: Non-Seoul Metropolitan Area MSE Summary for Single and Multiple Models, Table S8: Domestic Coefficients from Single Index Model, Table S9: Domestic Coefficients from Multiple Indices Model, Table S10: Domestic MSE Summary for Single and Multiple Models.
