# Supplementary material for: Statistical Estimation of Effects of Implemented Government Policies on COVID-19 Situation in South Korea
Source: Int J Environ Res Public Health. 2021 Feb 22;18(4):2144. doi: 10.3390/ijerph18042144 (PMC7926904; doi:10.3390/ijerph18042144)
Supplement: Supplementary file 1 [file ijerph-18-02144-s001.pdf]

Supplementary Materials

OxCGRT data

To adjust the differences between policies, each variable was divided by possible maximum level of each data and multiplied by 100 thus having range between 0 to 100. If the policy has flag, which indicates whether the policy was implemented generally in a country or locally, it was treated with additional level of 1, for the policy while increasing maximum level too by 1.

$$Index = \frac{policy\ level}{\max level} \text{ (without flag)} \qquad \text{(S1)}$$

$$Index = \frac{policy\ level + flag}{\max level + 1} \text{ (with flag)} \qquad \text{(S2)}$$

Table S1: Summary of Policy Variables Used for Analysis. The 13 government implemented policies recorded on an ordinal scale that represents the strength of the policy.

ID	Policy name	Type	Variable information
C1	School closing	Ordinal	Record closings of schools and universities<br>0 - no measures<br>1 - recommend closing or all schools open with alterations resulting in significant differences compared to non-Covid-19 operations<br>2 - require closing (only some levels or categories, e.g. just high school, or just public schools)<br>3 - require closing all levels
C2	Workplace closing	Ordinal	Record closings of workplaces<br>0 - no measures<br>1 - recommend closing (or recommend work from home)<br>2 - require closing (or work from home) for some sectors or categories of workers<br>3 - require closing (or work from home) for all-but-essential workplaces (e.g. grocery stores, doctors)
C3	Cancel public events	Ordinal	Record cancelling public events<br>0 - no measures<br>1 - recommend cancelling<br>2 - require cancelling

C4	Restrictions on gatherings	Ordinal	Record limits on private gatherings<br>0 - no restrictions<br>1 - restrictions on very large gatherings (the limit is above 1000 people)<br>2 - restrictions on gatherings between 101-1000 people<br>3 - restrictions on gatherings between 11-100 people<br>4 - restrictions on gatherings of 10 people or less
C5	Close public transport	Ordinal	Record closing of public transport<br>0 - no measures<br>1 - recommend closing (or significantly reduce volume/route/means of transport available)<br>2 - require closing (or prohibit most citizens from using it)
C6	Stay at home requirements	Ordinal	Record orders to "shelter-in-place" and otherwise confine to the home<br>0 - no measures<br>1 - recommend not leaving house<br>2 - require not leaving house with exceptions for daily exercise, grocery shopping, and 'essential' trips<br>3 - require not leaving house with minimal exceptions (e.g. allowed to leave once a week, or only one person can leave at a time, etc.)
C7	Restrictions on internal movement	Ordinal	Record restrictions on internal movement between cities/regions<br>0 - no measures<br>1 - recommend not to travel between regions/cities<br>2 - internal movement restrictions in place
C8	International travel controls	Ordinal	Record restrictions on international travel for foreign travelers<br>0 - no restrictions<br>1 - screening arrivals<br>2 - quarantine arrivals from some or all regions<br>3 - ban arrivals from some regions<br>4 - ban on all regions or total border closure

E1	Income support	Ordinal	Record if the government is providing direct cash payments to people who lose their jobs or cannot work.<br>Note: only includes payments to firms if explicitly linked to payroll/salaries<br>0 - no income support<br>1 - government is replacing less than 50% of lost salary (or if a flat sum, it is less than 50% median salary)<br>2 - government is replacing 50% or more of lost salary (or if a flat sum, it is greater than 50% median salary)
E2	Debt/contract relief	Ordinal	Record if the government is freezing financial obligations for households (e.g. stopping loan repayments, preventing services like water from stopping, or banning evictions)<br>0 - no debt/contract relief<br>1 - narrow relief, specific to one kind of contract<br>2 - broad debt/contract relief
H1	Public information campaigns	Ordinal	Record presence of public info campaigns<br>0 - no Covid-19 public information campaign<br>1 - public officials urging caution about Covid-19<br>2- coordinated public information campaign (e.g. across traditional and social media)
H2	Testing policy	Ordinal	Record government policy on who has access to testing<br>0 - no testing policy<br>1 - only those who both (a) have symptoms AND (b) meet specific criteria (e.g. key workers, admitted to hospital, came into contact with a known case, returned from overseas)<br>2 - testing of anyone showing Covid-19 symptoms
H3	Contact tracing	Ordinal	Record government policy on contact tracing after a positive diagnosis<br>0 - no contact tracing<br>1 - limited contact tracing; not done for all cases<br>2 - comprehensive contact tracing; done for all identified cases

Table S2: Seoul Metropolitan Area Coefficients from Single Index Model

Lag	Model Index	Intercept	Segment 1		Segment 2		Segment 3		Segment 4		Segment 5		Index
		b0	b11	b21	b12	b22	b13	b23	b14	b24	b15	b25	
0	Without Index	-50.599	-2.897	33.757	2.468	-6.274	0.224	0.349	0.034	1.315	0.053	0.245	NA
	Closing Index	-51.650	-3.318	35.851	2.872	-8.016	0.265	0.530	-0.009	1.310	0.098	0.233	0.069
	Restriction Index	-42.023	-2.463	28.336	2.082	-5.061	0.185	0.813	0.032	1.487	0.055	0.266	0.012
	Economic Index	-50.959	-3.011	34.378	2.501	-5.661	0.290	0.618	0.040	1.345	0.054	0.260	0.035
	Health Index	-50.856	-2.615	32.996	2.189	-5.805	0.223	0.355	0.033	1.315	0.052	0.244	-0.025
	Stringency Index	-30.961	-2.209	22.480	1.880	-4.127	0.162	1.251	0.013	1.543	0.083	0.305	0.062
	Korean Government Index	-45.008	-2.600	29.970	2.229	-5.749	0.190	0.394	0.019	1.169	0.028	0.627	0.014
1	Without Index	-50.599	-2.897	33.757	2.468	-6.274	0.224	0.349	0.034	1.315	0.053	0.245	NA
	Closing Index	-52.173	-3.155	35.456	2.717	-7.960	0.258	0.489	-0.006	1.321	0.097	0.200	0.061
	Restriction Index	-42.955	-2.515	28.943	2.126	-5.178	0.189	0.813	0.033	1.486	0.055	0.264	0.012
	Economic Index	-52.469	-3.098	35.376	2.576	-5.865	0.297	0.589	0.042	1.346	0.054	0.264	0.036
	Health Index	-50.645	-3.238	34.907	2.806	-6.968	0.225	0.341	0.034	1.316	0.053	0.247	0.029
	Stringency Index	-34.608	-2.291	24.432	1.943	-4.619	0.173	1.164	0.017	1.533	0.083	0.276	0.056
	Korean Government Index	-46.595	-2.681	31.029	2.294	-5.900	0.200	0.387	0.023	1.224	0.035	0.534	0.010
2	Without Index	-50.599	-2.897	33.757	2.468	-6.274	0.224	0.349	0.034	1.315	0.053	0.245	NA
	Closing Index	-51.921	-3.086	35.087	2.653	-7.935	0.255	0.473	-0.007	1.333	0.099	0.169	0.061
	Restriction Index	-43.162	-2.529	29.091	2.136	-5.193	0.190	0.856	0.034	1.502	0.056	0.261	0.012
	Economic Index	-54.065	-3.192	36.439	2.656	-6.064	0.306	0.565	0.043	1.348	0.054	0.268	0.037
	Health Index	-51.495	-3.204	35.199	2.770	-7.148	0.225	0.338	0.035	1.316	0.053	0.247	0.028
	Stringency Index	-35.691	-2.289	24.907	1.935	-4.808	0.175	1.200	0.017	1.556	0.088	0.254	0.058
	Korean Government Index	-47.336	-2.717	31.517	2.323	-5.972	0.204	0.386	0.024	1.251	0.039	0.491	0.008
3	Without Index	-50.599	-2.897	33.757	2.468	-6.274	0.224	0.349	0.034	1.315	0.053	0.245	NA
	Closing Index	-51.087	-2.962	34.233	2.538	-7.563	0.246	0.442	-0.002	1.339	0.093	0.156	0.050
	Restriction Index	-42.390	-2.495	28.622	2.103	-5.063	0.187	0.977	0.036	1.547	0.058	0.258	0.015
	Economic Index	-55.017	-3.234	37.014	2.700	-6.270	0.303	0.508	0.043	1.344	0.054	0.268	0.033
	Health Index	-54.356	-3.379	37.030	2.941	-8.282	0.227	0.325	0.036	1.316	0.053	0.249	0.052
	Stringency Index	-37.045	-2.288	25.491	1.927	-5.008	0.178	1.215	0.018	1.573	0.092	0.231	0.059

	Korean Government Index	-48.371	-2.771	32.214	2.367	-6.069	0.210	0.378	0.026	1.278	0.044	0.414	0.006
4	Without Index	-50.599	-2.897	33.757	2.468	-6.274	0.224	0.349	0.034	1.315	0.053	0.245	NA
	Closing Index	-50.326	-2.868	33.521	2.450	-7.179	0.237	0.417	0.004	1.341	0.086	0.156	0.039
	Restriction Index	-42.259	-2.493	28.558	2.097	-5.021	0.186	1.074	0.038	1.581	0.060	0.249	0.017
	Economic Index	-56.189	-3.297	37.771	2.757	-6.452	0.306	0.472	0.044	1.343	0.055	0.270	0.033
	Health Index	-60.799	-3.923	41.691	3.470	-11.133	0.232	0.291	0.038	1.317	0.054	0.256	0.118
	Stringency Index	-38.205	-2.326	26.145	1.957	-5.148	0.183	1.214	0.020	1.584	0.095	0.209	0.058
	Korean Government Index	-49.360	-2.826	32.891	2.410	-6.160	0.216	0.368	0.029	1.299	0.049	0.337	0.003
5	Without Index	-50.599	-2.897	33.757	2.468	-6.274	0.224	0.349	0.034	1.315	0.053	0.245	NA
	Closing Index	-49.511	-2.818	32.971	2.404	-6.893	0.232	0.403	0.008	1.342	0.082	0.150	0.033
	Restriction Index	-44.080	-2.586	29.714	2.179	-5.272	0.194	1.006	0.040	1.551	0.061	0.237	0.016
	Economic Index	-56.851	-3.325	38.167	2.788	-6.600	0.303	0.428	0.044	1.341	0.055	0.270	0.030
	Health Index	-44.652	-2.523	29.955	2.103	-3.794	0.221	0.377	0.032	1.316	0.052	0.241	-0.055
	Stringency Index	-39.982	-2.408	27.239	2.027	-5.332	0.190	1.156	0.022	1.573	0.095	0.184	0.054
	Korean Government Index	-51.206	-2.933	34.184	2.498	-6.331	0.227	0.338	0.036	1.322	0.054	0.202	-0.002
6	Without Index	-50.599	-2.897	33.757	2.468	-6.274	0.224	0.349	0.034	1.315	0.053	0.245	NA
	Closing Index	-49.247	-2.806	32.811	2.391	-6.536	0.226	0.382	0.017	1.334	0.071	0.172	0.020
	Restriction Index	-45.584	-2.663	30.667	2.246	-5.477	0.201	0.960	0.041	1.530	0.062	0.225	0.014
	Economic Index	-56.869	-3.313	38.123	2.785	-6.690	0.295	0.385	0.043	1.336	0.055	0.268	0.026
	Health Index	-39.653	-2.350	27.338	1.925	-1.554	0.226	0.397	0.032	1.320	0.052	0.241	-0.106
	Stringency Index	-42.575	-2.530	28.842	2.134	-5.590	0.200	1.030	0.025	1.535	0.092	0.166	0.046
	Korean Government Index	-52.664	-3.021	35.220	2.571	-6.471	0.236	0.309	0.041	1.330	0.055	0.109	-0.006
7	Without Index	-50.599	-2.897	33.757	2.468	-6.274	0.224	0.349	0.034	1.315	0.053	0.245	NA
	Closing Index	-49.577	-2.831	33.051	2.411	-6.348	0.224	0.366	0.025	1.326	0.062	0.201	0.010
	Restriction Index	-46.575	-2.714	31.300	2.289	-5.602	0.206	0.976	0.043	1.530	0.064	0.213	0.015
	Economic Index	-57.756	-3.361	38.695	2.828	-6.822	0.298	0.356	0.044	1.336	0.055	0.269	0.027
	Health Index	-42.844	-2.487	29.122	2.058	-2.668	0.228	0.384	0.033	1.320	0.052	0.244	-0.083
	Stringency Index	-44.289	-2.610	29.903	2.204	-5.770	0.207	0.963	0.027	1.516	0.091	0.148	0.041
	Korean Government Index	-54.000	-3.103	36.168	2.639	-6.607	0.244	0.276	0.046	1.330	0.054	0.043	-0.009
8	Without Index	-50.599	-2.897	33.757	2.468	-6.274	0.224	0.349	0.034	1.315	0.053	0.245	NA

	Closing Index	-50.958	-2.920	34.003	2.488	-6.272	0.224	0.344	0.036	1.313	0.050	0.258	-0.003
	Restriction Index	-48.016	-2.785	32.203	2.351	-5.805	0.212	0.910	0.043	1.500	0.064	0.204	0.013
	Economic Index	-60.027	-3.498	40.221	2.940	-7.068	0.315	0.325	0.046	1.339	0.055	0.275	0.032
	Health Index	-45.600	-2.573	30.524	2.141	-3.610	0.228	0.373	0.034	1.319	0.052	0.245	-0.063
	Stringency Index	-46.444	-2.709	31.224	2.292	-5.970	0.214	0.817	0.029	1.468	0.084	0.151	0.032
	Korean Government Index	-56.620	-3.242	37.917	2.755	-6.886	0.258	0.215	0.055	1.325	0.051	-0.055	-0.015
9	Without Index	-50.599	-2.897	33.757	2.468	-6.274	0.224	0.349	0.034	1.315	0.053	0.245	NA
	Closing Index	-51.391	-2.947	34.296	2.512	-6.293	0.224	0.340	0.038	1.309	0.048	0.271	-0.005
	Restriction Index	-49.160	-2.841	32.917	2.400	-5.965	0.218	0.861	0.044	1.477	0.064	0.197	0.012
	Economic Index	-61.681	-3.593	41.312	3.020	-7.275	0.325	0.285	0.048	1.339	0.056	0.279	0.034
	Health Index	-46.614	-2.585	30.964	2.140	-3.240	0.236	0.368	0.036	1.323	0.053	0.250	-0.077
	Stringency Index	-47.706	-2.766	31.995	2.343	-6.091	0.219	0.738	0.031	1.442	0.080	0.156	0.027
	Korean Government Index	-58.758	-3.304	39.080	2.804	-7.119	0.265	0.167	0.060	1.310	0.045	-0.081	-0.018
10	Without Index	-50.599	-2.897	33.757	2.468	-6.274	0.224	0.349	0.034	1.315	0.053	0.245	NA
	Closing Index	-52.436	-3.011	35.004	2.569	-6.359	0.226	0.330	0.043	1.302	0.043	0.299	-0.009
	Restriction Index	-50.106	-2.885	33.498	2.441	-6.110	0.222	0.765	0.043	1.439	0.063	0.194	0.010
	Economic Index	-62.849	-3.656	42.066	3.075	-7.442	0.330	0.243	0.049	1.338	0.056	0.281	0.036
	Health Index	-48.068	-2.642	31.722	2.195	-3.764	0.236	0.361	0.036	1.323	0.053	0.250	-0.065
	Stringency Index	-48.871	-2.818	32.701	2.390	-6.184	0.222	0.627	0.032	1.406	0.072	0.174	0.019
	Korean Government Index	-60.508	-3.314	39.815	2.807	-7.292	0.269	0.128	0.063	1.292	0.038	-0.071	-0.020

Table S3: Seoul Metropolitan Area Coefficients from Multiple Indices Model

Lag	b0	b11	b21	b12	b22	b13	b23	b14	b24	b15	b25	Closing Index	Restriction Index	Economic Index	Health Index	Korean Government Index
0	-40.004	-1.975	25.989	1.566	-4.409	0.250	1.138	-0.018	1.280	0.069	0.693	0.062	0.009	0.029	-0.074	0.016
1	-44.066	-2.495	29.397	2.056	-5.627	0.264	1.042	-0.008	1.350	0.075	0.551	0.054	0.008	0.029	-0.029	0.011
2	-43.251	-2.243	28.225	1.794	-4.458	0.271	1.066	-0.006	1.407	0.082	0.479	0.053	0.009	0.031	-0.062	0.010
3	-37.954	-1.941	24.841	1.513	-2.557	0.247	1.162	0.002	1.494	0.080	0.423	0.041	0.013	0.025	-0.090	0.008
4	-41.242	-2.358	27.696	1.919	-4.087	0.243	1.181	0.014	1.554	0.080	0.357	0.030	0.016	0.023	-0.042	0.006
5	-39.553	-2.346	27.282	1.864	-1.808	0.273	1.027	0.024	1.546	0.085	0.177	0.027	0.012	0.025	-0.101	0.000
6	-41.510	-2.551	29.070	2.035	-1.231	0.289	0.893	0.039	1.531	0.079	0.085	0.017	0.011	0.023	-0.119	-0.004
7	-47.632	-2.851	32.805	2.303	-2.517	0.303	0.822	0.053	1.527	0.073	0.032	0.009	0.011	0.024	-0.096	-0.008
8	-57.221	-3.295	38.655	2.679	-3.903	0.344	0.616	0.071	1.484	0.063	-0.041	0.002	0.009	0.031	-0.082	-0.014
9	-63.741	-3.511	42.258	2.840	-4.166	0.381	0.464	0.079	1.456	0.060	-0.085	0.004	0.008	0.035	-0.096	-0.018
10	-68.929	-3.667	44.932	2.976	-5.046	0.393	0.307	0.085	1.409	0.051	-0.070	0.002	0.006	0.037	-0.085	-0.019

Table S4: Seoul Metropolitan Area MSE Summary for Single and Multiple Indices Models

Lag	Single Index Model												Multiple Indices Model	
	Closing Index		Restriction Index		Economic Index		Health Index		Stringency Index		Korean Government Index			
	Train MSE	Test MSE	Train MSE	Test MSE	Train MSE	Test MSE	Train MSE	Test MSE	Train MSE	Test MSE	Train MSE	Test MSE	Train MSE	Test MSE
0	661.3	3614.0	923.6	6992.5	915.3	7035.3	938.9	6922.0	711.2	3956.4	899.6	7680.6	628.5	4729.4
1	715.9	3780.3	918.3	6991.6	913.0	7062.9	935.8	6944.5	753.8	4121.4	925.6	7764.4	704.1	5118.7
2	707.4	3657.3	912.3	6961.9	908.5	7094.3	935.4	6947.8	738.1	3897.7	925.0	7836.1	691.8	5188.7
3	752.5	4125.1	895.7	6901.3	911.2	7098.8	932.6	6968.2	736.8	3779.1	930.2	7475.1	733.4	5425.0
4	807.9	4703.5	876.5	6797.5	910.2	7115.7	925.3	7022.1	749.0	3779.3	935.0	7169.0	769.2	5632.4
5	839.9	4996.0	883.6	6703.7	911.4	7117.7	942.8	6894.2	771.6	3902.6	936.2	6853.9	800.4	5294.7

6	888.6	5714.7	885.3	6618.7	914.3	7103.9	942.6	6895.6	811.5	4285.5	922.1	6816.9	824.0	5740.9
7	918.2	6307.7	878.5	6507.8	912.1	7116.7	939.9	6915.1	833.3	4481.6	899.4	6987.6	816.2	6290.8
8	940.3	7091.1	883.8	6457.8	904.2	7164.2	938.3	6927.0	876.8	5042.6	843.5	7402.2	773.7	7184.4
9	941.1	7210.4	887.7	6413.6	900.2	7193.6	933.3	6963.3	891.2	5441.3	813.7	7957.8	732.8	7722.9
10	940.3	7429.6	898.0	6409.1	897.5	7210.0	932.5	6968.8	909.8	5958.1	793.6	8535.0	719.8	8425.4

The train and test MSE of Without index model: 937.7, 6930.6

Table S5: Non-Seoul Metropolitan Area Coefficients from Single Index Model

Lag	Model Index	Intercept	Segment 1		Segment 2		Segment 3		Segment 4		Segment 5		Index
		b0	b11	b21	b12	b22	b13	b23	b14	b24	b15	b25	
0	Without Index	-1.540	10.761	-87.990	-10.606	54.521	0.012	0.913	-0.078	0.906	0.062	-0.358	NA
	Closing Index	-1.540	3.708	-34.697	-3.799	24.975	0.147	0.745	-0.075	0.911	0.107	-0.253	0.058
	Restriction Index	-1.540	10.001	-83.011	-9.903	52.409	0.025	1.570	-0.062	1.119	0.069	-0.280	0.017
	Economic Index	-1.540	9.761	-85.656	-9.777	57.515	0.135	1.309	-0.058	0.947	0.066	-0.323	0.041
	Health Index	-1.540	16.302	-94.820	-16.112	58.115	-0.004	0.937	-0.084	0.905	0.060	-0.369	-0.649
	Stringency Index	-1.540	8.454	-72.576	-8.424	47.047	0.071	1.646	-0.063	1.078	0.093	-0.219	0.055
	Korean Government Index	-2.418	12.009	-96.393	-11.767	57.596	-0.037	1.016	-0.101	0.669	0.019	0.336	0.026
1	Without Index	-1.540	10.761	-87.990	-10.606	54.521	0.012	0.913	-0.078	0.906	0.062	-0.358	NA
	Closing Index	-1.540	3.869	-34.598	-3.941	24.099	0.134	0.690	-0.076	0.921	0.109	-0.287	0.055
	Restriction Index	-1.540	9.145	-76.242	-9.078	48.644	0.037	1.562	-0.056	1.132	0.071	-0.271	0.018
	Economic Index	-1.540	8.560	-78.457	-8.663	55.202	0.190	1.358	-0.047	0.957	0.068	-0.302	0.053
	Health Index	-1.540	14.836	-93.397	-14.654	57.375	0.000	0.933	-0.083	0.906	0.061	-0.367	-0.471
	Stringency Index	-1.540	7.155	-62.024	-7.166	40.988	0.087	1.574	-0.056	1.092	0.099	-0.229	0.056
	Korean Government Index	-1.540	11.359	-91.864	-11.140	55.218	-0.026	1.006	-0.098	0.714	0.023	0.335	0.025
2	Without Index	-1.540	10.761	-87.990	-10.606	54.521	0.012	0.913	-0.078	0.906	0.062	-0.358	NA
	Closing Index	-1.540	4.275	-36.259	-4.317	23.978	0.117	0.654	-0.083	0.932	0.112	-0.323	0.057
	Restriction Index	-1.540	8.606	-71.706	-8.554	45.949	0.042	1.536	-0.052	1.135	0.072	-0.274	0.018
	Economic Index	-1.540	7.633	-71.852	-7.776	52.011	0.211	1.276	-0.041	0.955	0.070	-0.291	0.056

	Health Index	-1.540	13.667	-92.273	-13.491	56.790	0.002	0.929	-0.082	0.906	0.061	-0.365	-0.328
	Stringency Index	-1.540	6.451	-55.796	-6.479	37.078	0.092	1.525	-0.053	1.106	0.104	-0.248	0.057
	Korean Government Index	-1.540	10.583	-85.800	-10.394	51.939	-0.013	0.985	-0.094	0.752	0.029	0.316	0.024
3	Without Index	-1.540	10.761	-87.990	-10.606	54.521	0.012	0.913	-0.078	0.906	0.062	-0.358	NA
	Closing Index	-1.540	5.423	-43.554	-5.407	26.907	0.088	0.653	-0.093	0.944	0.113	-0.364	0.057
	Restriction Index	-1.540	7.645	-63.879	-7.632	41.459	0.052	1.724	-0.043	1.219	0.078	-0.258	0.025
	Economic Index	-1.540	7.090	-67.412	-7.242	49.371	0.211	1.156	-0.040	0.948	0.070	-0.288	0.053
	Health Index	-1.540	12.885	-91.967	-12.711	56.636	0.003	0.929	-0.081	0.906	0.061	-0.365	-0.224
	Stringency Index	-1.540	5.788	-49.798	-5.834	33.124	0.096	1.598	-0.051	1.156	0.116	-0.264	0.068
	Korean Government Index	-1.540	9.710	-78.910	-9.555	48.205	0.002	0.950	-0.089	0.790	0.038	0.248	0.022
4	Without Index	-1.540	10.761	-87.990	-10.606	54.521	0.012	0.913	-0.078	0.906	0.062	-0.358	NA
	Closing Index	-1.540	7.445	-58.403	-7.355	34.739	0.048	0.710	-0.098	0.945	0.103	-0.397	0.046
	Restriction Index	-1.540	7.397	-61.189	-7.390	39.504	0.049	1.846	-0.039	1.269	0.082	-0.267	0.029
	Economic Index	-1.540	5.988	-59.932	-6.197	46.079	0.245	1.089	-0.032	0.949	0.072	-0.273	0.060
	Health Index	-1.540	12.321	-91.897	-12.146	56.609	0.004	0.929	-0.081	0.906	0.061	-0.364	-0.147
	Stringency Index	-1.540	6.375	-53.041	-6.387	33.998	0.076	1.615	-0.057	1.173	0.120	-0.304	0.071
	Korean Government Index	-1.540	8.884	-72.197	-8.757	44.382	0.014	0.918	-0.085	0.815	0.046	0.219	0.021
5	Without Index	-1.540	10.761	-87.990	-10.606	54.521	0.012	0.913	-0.078	0.906	0.062	-0.358	NA
	Closing Index	-1.540	9.058	-70.226	-8.907	40.886	0.019	0.762	-0.105	0.946	0.099	-0.440	0.041
	Restriction Index	-1.540	7.796	-63.362	-7.768	40.098	0.036	1.926	-0.041	1.289	0.085	-0.295	0.031
	Economic Index	-1.540	5.655	-56.991	-5.863	44.176	0.241	0.978	-0.032	0.942	0.073	-0.272	0.058
	Health Index	-1.540	11.509	-90.220	-11.343	55.718	0.007	0.923	-0.080	0.906	0.061	-0.362	-0.064
	Stringency Index	-1.540	7.393	-59.583	-7.356	36.649	0.051	1.665	-0.065	1.185	0.126	-0.371	0.074
	Korean Government Index	-1.540	9.307	-75.696	-9.180	46.774	0.018	0.895	-0.080	0.867	0.057	-0.061	0.012
6	Without Index	-1.540	10.761	-87.990	-10.606	54.521	0.012	0.913	-0.078	0.906	0.062	-0.358	NA
	Closing Index	-1.540	10.546	-82.927	-10.360	48.976	0.000	0.844	-0.100	0.932	0.085	-0.439	0.024
	Restriction Index	-1.540	8.653	-69.396	-8.588	42.997	0.020	1.888	-0.047	1.248	0.084	-0.337	0.030
	Economic Index	-1.540	5.057	-52.578	-5.288	41.912	0.252	0.884	-0.029	0.938	0.073	-0.266	0.060
	Health Index	-1.540	10.665	-87.648	-10.512	54.336	0.013	0.912	-0.078	0.906	0.062	-0.358	0.007
	Stringency Index	-1.540	8.914	-70.659	-8.814	42.351	0.023	1.581	-0.074	1.135	0.121	-0.438	0.064

	Korean Government Index	-1.540	9.230	-74.776	-9.103	46.086	0.017	0.886	-0.080	0.877	0.061	-0.091	0.011
	Without Index	-1.540	10.761	-87.990	-10.606	54.521	0.012	0.913	-0.078	0.906	0.062	-0.358	NA
	Closing Index	-1.540	11.185	-89.088	-10.991	53.386	-0.004	0.894	-0.094	0.922	0.075	-0.423	0.014
	Restriction Index	-1.540	9.838	-78.924	-9.722	48.402	0.008	1.569	-0.060	1.108	0.077	-0.375	0.020
7	Economic Index	-1.540	3.816	-44.164	-4.112	38.222	0.291	0.787	-0.020	0.938	0.076	-0.250	0.068
	Health Index	-1.540	9.749	-83.623	-9.614	52.151	0.021	0.893	-0.074	0.906	0.063	-0.352	0.063
	Stringency Index	-1.540	10.272	-81.419	-10.122	48.605	0.004	1.402	-0.081	1.055	0.106	-0.466	0.045
	Korean Government Index	-1.540	9.343	-75.281	-9.210	46.133	0.014	0.885	-0.081	0.886	0.064	-0.119	0.011
	Without Index	-1.540	10.761	-87.990	-10.606	54.521	0.012	0.913	-0.078	0.906	0.062	-0.358	NA
	Closing Index	-1.540	11.228	-90.510	-11.045	55.040	0.000	0.917	-0.087	0.914	0.068	-0.397	0.006
	Restriction Index	-1.540	10.543	-84.889	-10.399	51.966	0.004	1.348	-0.069	1.022	0.072	-0.389	0.013
8	Economic Index	-1.540	2.779	-36.975	-3.126	34.927	0.320	0.674	-0.014	0.936	0.077	-0.237	0.075
	Health Index	-1.540	8.485	-76.582	-8.382	48.293	0.035	0.857	-0.069	0.906	0.064	-0.341	0.114
	Stringency Index	-1.540	11.086	-88.330	-10.909	52.954	-0.004	1.257	-0.084	0.997	0.092	-0.465	0.030
	Korean Government Index	-1.540	9.154	-72.501	-9.011	43.612	0.004	0.885	-0.087	0.887	0.070	-0.045	0.016
	Without Index	-1.540	10.761	-87.990	-10.606	54.521	0.012	0.913	-0.078	0.906	0.062	-0.358	NA
	Closing Index	-1.540	9.477	-80.018	-9.386	51.697	0.039	0.883	-0.059	0.891	0.052	-0.281	-0.011
	Restriction Index	-1.540	10.856	-88.081	-10.699	54.181	0.006	1.102	-0.075	0.950	0.066	-0.380	0.005
9	Economic Index	-1.540	1.759	-29.870	-2.155	31.643	0.349	0.549	-0.008	0.932	0.079	-0.225	0.082
	Health Index	-1.540	7.473	-68.330	-7.406	43.716	0.050	0.812	-0.062	0.905	0.066	-0.329	0.109
	Stringency Index	-1.540	11.093	-89.825	-10.923	54.941	0.003	1.025	-0.081	0.933	0.071	-0.398	0.009
	Korean Government Index	-1.540	9.748	-76.396	-9.577	45.270	-0.009	0.907	-0.093	0.897	0.074	-0.077	0.017
	Without Index	-1.540	10.761	-87.990	-10.606	54.521	0.012	0.913	-0.078	0.906	0.062	-0.358	NA
	Closing Index	-1.540	7.828	-68.633	-7.807	46.546	0.068	0.814	-0.042	0.875	0.046	-0.210	-0.019
	Restriction Index	-1.540	10.756	-87.963	-10.601	54.513	0.012	0.910	-0.078	0.905	0.062	-0.358	0.000
10	Economic Index	-1.540	1.388	-26.963	-1.794	30.011	0.353	0.434	-0.007	0.926	0.079	-0.223	0.084
	Health Index	-1.540	6.712	-60.804	-6.676	39.464	0.063	0.768	-0.057	0.905	0.067	-0.319	0.084
	Stringency Index	-1.540	10.625	-87.118	-10.475	54.190	0.015	0.883	-0.077	0.900	0.060	-0.347	-0.002
	Korean Government Index	-1.540	10.619	-83.829	-10.430	49.928	-0.013	0.931	-0.093	0.907	0.072	-0.194	0.012

Table S6: Non-Seoul Metropolitan Area Coefficients from Multiple Indices Model

Lag	b0	b11	b21	b12	b22	b13	b23	b14	b24	b15	b25	Closing Index	Restriction Index	Economic Index	Health Index	Korean Government Index
0	-2.702	14.315	-79.214	-14.262	50.616	0.080	1.728	-0.091	0.747	0.035	0.666	0.030	0.012	0.028	-0.713	0.035
1	-1.540	7.905	-49.823	-8.022	35.466	0.174	1.436	-0.072	0.786	0.054	0.572	0.036	0.007	0.038	-0.374	0.030
2	-1.540	2.032	-19.073	-2.256	17.385	0.229	0.980	-0.068	0.772	0.077	0.510	0.052	0.001	0.038	-0.078	0.028
3	-1.541	0.450	-10.902	-0.659	10.896	0.197	1.111	-0.066	0.884	0.084	0.555	0.048	0.010	0.029	0.048	0.030
4	-1.541	0.521	-12.306	-0.733	11.710	0.184	1.396	-0.052	1.017	0.085	0.540	0.031	0.018	0.030	0.076	0.031
5	-1.540	1.782	-23.161	-1.956	17.934	0.161	1.505	-0.044	1.134	0.096	0.164	0.021	0.022	0.029	0.104	0.019
6	-1.540	1.984	-26.880	-2.181	21.532	0.163	1.713	-0.022	1.211	0.090	0.088	-0.001	0.029	0.030	0.118	0.016
7	-1.540	0.787	-21.642	-1.074	21.178	0.234	1.314	-0.013	1.108	0.090	0.011	-0.003	0.019	0.049	0.134	0.013
8	-1.566	-2.045	-2.289	1.678	10.243	0.277	0.988	-0.010	1.046	0.097	0.134	-0.001	0.013	0.057	0.189	0.019
9	-12.540	-3.596	15.184	3.111	2.616	0.328	0.883	0.021	1.056	0.090	0.203	-0.019	0.015	0.061	0.161	0.019
10	-23.070	-3.598	21.664	3.051	0.764	0.363	0.647	0.037	1.019	0.084	0.128	-0.026	0.013	0.065	0.112	0.013

Table S7: Non-Seoul Metropolitan Area MSE Summary for Single and Multiple Models

Lag	Single Index Model												Multiple Indices Model	
	Closing Index		Restriction Index		Economic Index		Health Index		Stringency Index		Korean Government Index			
	Train MSE	Test MSE	Train MSE	Test MSE	Train MSE	Test MSE	Train MSE	Test MSE	Train MSE	Test MSE	Train MSE	Test MSE	Train MSE	Test MSE
0	1745.5	519.0	1391.2	983.9	1246.7	945.5	1465.2	858.7	1387.8	508.5	1542.9	1020.1	1371.2	889.6
1	1668.8	491.9	1398.4	1004.8	1138.4	987.1	1474.4	863.5	1393.1	486.7	1565.1	1317.3	1403.9	1188.1
2	1297.5	430.6	1391.0	997.6	1108.2	1010.5	1481.7	867.4	1266.2	449.3	1594.0	1464.9	1241.2	1179.5
3	942.4	384.4	1160.7	1008.8	1157.2	1019.0	1483.8	868.5	963.0	343.0	1619.5	1338.8	926.6	1146.5
4	1010.3	434.0	915.5	975.8	1127.7	1049.7	1484.6	868.8	846.5	289.3	1647.0	1233.1	819.8	1173.9
5	997.8	420.5	790.3	911.4	1188.9	1052.8	1495.4	874.3	713.7	209.8	1615.7	1014.8	715.1	924.5

6	1379.2	551.0	821.0	836.6	1210.7	1065.8	1511.7	882.6	1012.4	215.5	1573.6	945.6	736.1	1020.2
7	1448.2	667.0	1346.3	799.5	1194.9	1099.9	1536.7	895.3	1324.8	316.7	1510.5	877.3	1169.2	1018.4
8	1468.3	769.9	1458.8	794.3	1195.9	1126.6	1579.6	917.1	1396.7	448.2	1246.7	770.0	1046.0	942.8
9	1450.1	1083.9	1539.1	829.9	1199.4	1152.3	1628.4	942.2	1552.3	739.9	1010.2	664.3	852.6	1137.3
10	1382.4	1226.0	1508.4	882.7	1230.9	1157.7	1671.7	964.2	1493.5	916.5	1159.0	663.0	1013.2	1243.7

The train and test MSE of Without policy: 1509.6, 881.5

Table S8: Domestic Coefficients from Single Index Model

Lag	Model Index	Intercept	Segment 1		Segment 2		Segment 3		Segment 4		Segment 5		Index
		b0	b11	b21	b12	b22	b13	b23	b14	b24	b15	b25	
0	Without Index	-7.058	-2.044	9.829	1.693	4.294	0.220	0.956	0.014	1.236	0.056	0.109	NA
	Closing Index	-16.305	-1.784	14.158	1.464	-0.951	0.232	0.952	-0.042	1.211	0.105	0.097	0.077
	Restriction Index	-7.224	-2.005	9.834	1.635	4.143	0.213	1.617	0.024	1.503	0.063	0.175	0.018
	Economic Index	-12.807	-3.033	15.967	2.503	4.769	0.347	1.289	0.034	1.285	0.060	0.153	0.040
	Health Index	0.296	-0.246	1.434	-0.066	8.826	0.203	1.013	0.008	1.233	0.055	0.094	-0.173
	Stringency Index	-8.823	-2.136	11.165	1.755	2.909	0.230	1.791	0.011	1.481	0.092	0.219	0.065
	Korean Government Index	-6.150	-1.676	7.899	1.385	3.672	0.182	0.948	-0.002	1.043	0.025	0.633	0.019
1	Without Index	-7.058	-2.044	9.829	1.693	4.294	0.220	0.956	0.014	1.236	0.056	0.109	NA
	Closing Index	-12.694	-1.581	11.684	1.286	-0.142	0.216	0.921	-0.042	1.222	0.104	0.053	0.071
	Restriction Index	-7.389	-1.957	9.810	1.589	3.981	0.209	1.672	0.024	1.531	0.064	0.175	0.020
	Economic Index	-16.582	-3.352	19.058	2.768	4.225	0.384	1.316	0.040	1.295	0.062	0.166	0.051
	Health Index	-1.159	-0.814	3.463	0.491	7.788	0.208	1.003	0.010	1.234	0.055	0.098	-0.117
	Stringency Index	-8.264	-2.007	10.480	1.640	2.849	0.221	1.781	0.009	1.497	0.096	0.187	0.066
	Korean Government Index	-6.706	-1.664	8.252	1.371	3.451	0.184	0.953	-0.002	1.081	0.027	0.615	0.017
2	Without Index	-7.058	-2.044	9.829	1.693	4.294	0.220	0.956	0.014	1.236	0.056	0.109	NA
	Closing Index	-8.931	-1.392	9.112	1.120	0.687	0.203	0.909	-0.045	1.234	0.106	0.013	0.069
	Restriction Index	-7.104	-1.890	9.456	1.527	3.960	0.203	1.715	0.025	1.552	0.065	0.166	0.021
	Economic Index	-19.966	-3.512	21.427	2.900	3.485	0.402	1.271	0.043	1.297	0.062	0.173	0.056

	Health Index	-2.542	-1.227	5.121	0.895	6.929	0.212	0.995	0.011	1.236	0.055	0.101	-0.076
	Stringency Index	-6.932	-1.860	9.272	1.509	3.028	0.211	1.802	0.007	1.520	0.101	0.153	0.069
	Korean Government Index	-6.895	-1.608	8.218	1.320	3.268	0.181	0.960	-0.005	1.101	0.028	0.633	0.018
3	Without Index	-7.058	-2.044	9.829	1.693	4.294	0.220	0.956	0.014	1.236	0.056	0.109	NA
	Closing Index	-5.394	-1.238	6.653	0.984	1.749	0.192	0.911	-0.045	1.246	0.104	-0.015	0.063
	Restriction Index	-6.353	-1.783	8.706	1.426	4.049	0.195	1.882	0.027	1.625	0.068	0.162	0.026
	Economic Index	-22.297	-3.529	22.754	2.914	2.797	0.403	1.183	0.043	1.292	0.063	0.174	0.057
	Health Index	-4.236	-1.612	7.019	1.272	5.891	0.215	0.980	0.012	1.236	0.056	0.104	-0.040
	Stringency Index	-4.982	-1.686	7.571	1.352	3.398	0.200	1.873	0.004	1.559	0.109	0.119	0.076
	Korean Government Index	-6.610	-1.565	7.969	1.280	3.273	0.179	0.969	-0.006	1.127	0.032	0.609	0.017
4	Without Index	-7.058	-2.044	9.829	1.693	4.294	0.220	0.956	0.014	1.236	0.056	0.109	NA
	Closing Index	-3.285	-1.218	5.257	0.962	2.938	0.189	0.928	-0.036	1.253	0.096	-0.018	0.050
	Restriction Index	-5.148	-1.668	7.637	1.320	4.321	0.186	1.995	0.028	1.670	0.071	0.144	0.029
	Economic Index	-26.269	-3.676	25.308	3.031	1.983	0.423	1.122	0.046	1.293	0.063	0.180	0.063
	Health Index	-6.935	-2.029	9.714	1.678	4.361	0.220	0.957	0.014	1.236	0.056	0.108	-0.001
	Stringency Index	-3.016	-1.517	5.794	1.199	3.966	0.190	1.884	0.001	1.574	0.113	0.082	0.077
	Korean Government Index	-5.939	-1.523	7.520	1.242	3.380	0.176	0.980	-0.007	1.149	0.037	0.578	0.017
5	Without Index	-7.058	-2.044	9.829	1.693	4.294	0.220	0.956	0.014	1.236	0.056	0.109	NA
	Closing Index	-2.144	-1.231	4.471	0.968	3.805	0.189	0.950	-0.029	1.257	0.091	-0.026	0.041
	Restriction Index	-3.967	-1.584	6.633	1.244	4.685	0.181	2.008	0.028	1.666	0.073	0.116	0.029
	Economic Index	-28.314	-3.659	26.310	3.014	1.397	0.422	1.026	0.045	1.287	0.063	0.180	0.064
	Health Index	-10.005	-2.340	12.383	1.980	2.799	0.223	0.931	0.016	1.236	0.057	0.112	0.028
	Stringency Index	-1.522	-1.375	4.302	1.069	4.533	0.183	1.879	0.000	1.577	0.116	0.033	0.076
	Korean Government Index	-5.751	-1.650	7.883	1.351	3.739	0.188	0.980	-0.002	1.190	0.047	0.404	0.011
6	Without Index	-7.058	-2.044	9.829	1.693	4.294	0.220	0.956	0.014	1.236	0.056	0.109	NA
	Closing Index	-3.135	-1.532	5.917	1.235	4.457	0.200	0.965	-0.010	1.250	0.076	0.017	0.022
	Restriction Index	-3.235	-1.555	6.044	1.220	4.998	0.180	1.945	0.027	1.625	0.074	0.086	0.027
	Economic Index	-30.505	-3.657	27.413	3.008	0.839	0.424	0.934	0.045	1.282	0.063	0.180	0.066
	Health Index	-14.131	-2.598	15.472	2.229	0.920	0.226	0.898	0.017	1.236	0.057	0.116	0.055
	Stringency Index	-1.078	-1.360	3.884	1.057	4.981	0.184	1.743	0.001	1.524	0.111	-0.003	0.064

	Korean Government Index	-5.601	-1.699	7.974	1.392	3.918	0.193	0.985	0.001	1.209	0.052	0.328	0.009
7	Without Index	-7.058	-2.044	9.829	1.693	4.294	0.220	0.956	0.014	1.236	0.056	0.109	NA
	Closing Index	-5.239	-1.856	8.165	1.524	4.513	0.213	0.965	0.006	1.242	0.063	0.072	0.007
	Restriction Index	-3.378	-1.635	6.335	1.299	5.114	0.188	1.713	0.024	1.514	0.071	0.066	0.021
	Economic Index	-34.451	-3.751	29.677	3.078	0.081	0.441	0.844	0.048	1.280	0.064	0.185	0.072
	Health Index	-19.884	-2.839	19.283	2.462	-1.505	0.228	0.851	0.019	1.234	0.058	0.120	0.087
	Stringency Index	-1.432	-1.463	4.418	1.154	5.227	0.190	1.544	0.003	1.446	0.100	-0.012	0.048
	Korean Government Index	-5.610	-1.761	8.164	1.445	4.097	0.198	0.988	0.003	1.223	0.055	0.259	0.007
8	Without Index	-7.058	-2.044	9.829	1.693	4.294	0.220	0.956	0.014	1.236	0.056	0.109	NA
	Closing Index	-8.551	-2.168	11.086	1.804	4.052	0.224	0.946	0.020	1.232	0.052	0.135	-0.005
	Restriction Index	-3.901	-1.740	6.960	1.401	5.092	0.197	1.494	0.021	1.419	0.068	0.060	0.014
	Economic Index	-38.575	-3.838	31.965	3.140	-0.677	0.458	0.744	0.050	1.277	0.065	0.190	0.079
	Health Index	-25.881	-2.985	22.783	2.604	-3.937	0.227	0.798	0.020	1.230	0.058	0.122	0.118
	Stringency Index	-2.521	-1.638	5.697	1.318	5.202	0.199	1.336	0.007	1.367	0.086	0.008	0.030
	Korean Government Index	-5.403	-1.773	8.065	1.454	4.235	0.200	0.994	0.004	1.230	0.057	0.230	0.006
9	Without Index	-7.058	-2.044	9.829	1.693	4.294	0.220	0.956	0.014	1.236	0.056	0.109	NA
	Closing Index	-15.680	-2.602	16.411	2.188	2.756	0.241	0.886	0.040	1.211	0.034	0.244	-0.022
	Restriction Index	-4.965	-1.873	8.023	1.529	4.869	0.208	1.255	0.018	1.329	0.063	0.072	0.008
	Economic Index	-41.943	-3.879	33.702	3.165	-1.307	0.470	0.635	0.051	1.273	0.065	0.192	0.085
	Health Index	-28.650	-2.809	23.555	2.439	-4.971	0.219	0.761	0.019	1.225	0.058	0.119	0.124
	Stringency Index	-5.158	-1.904	8.230	1.564	4.706	0.213	1.084	0.012	1.279	0.066	0.070	0.010
	Korean Government Index	-5.566	-1.837	8.337	1.510	4.360	0.205	0.992	0.007	1.236	0.058	0.185	0.004
10	Without Index	-7.058	-2.044	9.829	1.693	4.294	0.220	0.956	0.014	1.236	0.056	0.109	NA
	Closing Index	-20.143	-2.777	19.324	2.343	1.876	0.246	0.829	0.050	1.192	0.025	0.306	-0.030
	Restriction Index	-6.431	-2.002	9.318	1.652	4.476	0.217	1.033	0.015	1.258	0.058	0.096	0.002
	Economic Index	-43.538	-3.844	34.317	3.130	-1.674	0.470	0.524	0.050	1.265	0.065	0.190	0.088
	Health Index	-29.191	-2.514	22.795	2.165	-5.279	0.206	0.732	0.016	1.218	0.057	0.112	0.121
	Stringency Index	-7.895	-2.097	10.493	1.742	4.109	0.222	0.904	0.015	1.220	0.052	0.125	-0.004
	Korean Government Index	-7.119	-2.051	9.886	1.699	4.288	0.220	0.954	0.014	1.236	0.056	0.106	0.000

Table S9: Domestic Coefficients from Multiple Indices Model

Lag	b0	b11	b21	b12	b22	b13	b23	b14	b24	b15	b25	Closing Index	Restriction Index	Economic Index	Health Index	Korean Government Index
0	-11.445	-1.036	9.576	0.682	2.577	0.255	1.463	-0.038	1.114	0.067	0.722	0.065	0.007	0.026	-0.101	0.021
1	-17.224	-1.708	14.290	1.306	0.578	0.283	1.434	-0.028	1.187	0.072	0.629	0.058	0.007	0.036	-0.037	0.018
2	-20.964	-2.039	16.939	1.632	-1.325	0.286	1.371	-0.029	1.223	0.078	0.592	0.057	0.007	0.039	0.015	0.018
3	-20.718	-2.042	16.622	1.663	-2.232	0.253	1.529	-0.025	1.337	0.079	0.592	0.048	0.014	0.034	0.052	0.018
4	-22.053	-2.178	17.646	1.782	-2.537	0.250	1.700	-0.011	1.443	0.077	0.590	0.033	0.019	0.036	0.072	0.019
5	-24.158	-2.451	19.689	2.020	-2.758	0.268	1.672	0.003	1.503	0.085	0.387	0.025	0.020	0.039	0.083	0.012
6	-25.497	-2.757	21.539	2.281	-2.143	0.283	1.715	0.026	1.549	0.077	0.349	0.006	0.023	0.042	0.077	0.010
7	-34.127	-3.330	27.748	2.775	-3.410	0.334	1.474	0.042	1.494	0.072	0.301	-0.003	0.018	0.053	0.086	0.007
8	-42.927	-3.723	33.284	3.120	-5.355	0.364	1.251	0.051	1.442	0.068	0.313	-0.009	0.015	0.060	0.106	0.007
9	-49.873	-3.898	37.227	3.246	-5.741	0.385	1.104	0.069	1.412	0.054	0.355	-0.024	0.014	0.064	0.089	0.006
10	-55.090	-3.927	39.759	3.250	-6.092	0.400	0.865	0.078	1.352	0.047	0.318	-0.029	0.012	0.065	0.075	0.002

Table S10: Domestic MSE Summary for Single and Multiple Models

Lag	Single Index Model												Multiple Index Model	
	Closing Index		Restriction Index		Economic Index		Health Index		Stringency Index		Korean Government Index			
	Train MSE	Test MSE	Train MSE	Test MSE	Train MSE	Test MSE	Train MSE	Test MSE	Train MSE	Test MSE	Train MSE	Test MSE	Train MSE	Test MSE
0	3166.0	5819.0	3725.8	14574.6	3755.5	14540.2	4234.3	13533.2	3156.7	7908.2	4314.2	15863.8	2938.4	9216.4
1	3089.1	5827.5	3598.7	14578.9	3522.3	14772.6	4262.6	13607.6	3005.8	7280.4	4332.5	16980.3	2840.0	10877.7
2	2654.2	5557.6	3483.5	14405.3	3370.7	14894.0	4286.6	13666.8	2690.7	6518.1	4278.9	18011.0	2335.4	11775.8
3	2465.1	5836.5	3081.8	14175.5	3384.3	14912.5	4310.7	13727.4	2242.4	5511.1	4206.2	17277.7	1977.7	12072.5
4	2907.5	7244.4	2780.0	13726.4	3269.9	15037.1	4339.0	13805.9	2170.9	5188.1	4126.5	16346.2	1982.9	12875.4
5	3275.1	8111.2	2783.7	13168.3	3323.0	15034.0	4361.5	13877.9	2247.8	4829.5	4184.8	14851.5	2142.7	11746.1

6	4032.0	10572.6	3021.8	12668.1	3326.6	15044.0	4377.9	13952.3	2951.0	5634.4	4175.7	14077.7	2487.3	13046.7
7	4289.6	12686.3	3775.5	12506.0	3247.3	15138.6	4384.2	14030.6	3689.7	7366.1	4188.0	13580.9	2952.8	13889.2
8	4346.4	14531.0	4138.6	12596.9	3176.8	15231.4	4359.2	14077.5	4115.7	9552.4	4139.3	13224.7	2999.0	14522.7
9	3991.9	16972.3	4370.5	12987.2	3143.2	15284.6	4278.7	14026.0	4397.8	12456.6	4166.4	13135.6	2712.1	16198.4
10	3660.4	17821.1	4379.2	13549.1	3169.0	15263.4	4162.3	13912.2	4286.5	14295.3	4344.7	13838.2	2723.7	16913.2

The train and test MSE of Without policy: 4340.1, 13809.1

SeoulMetro prediction plots

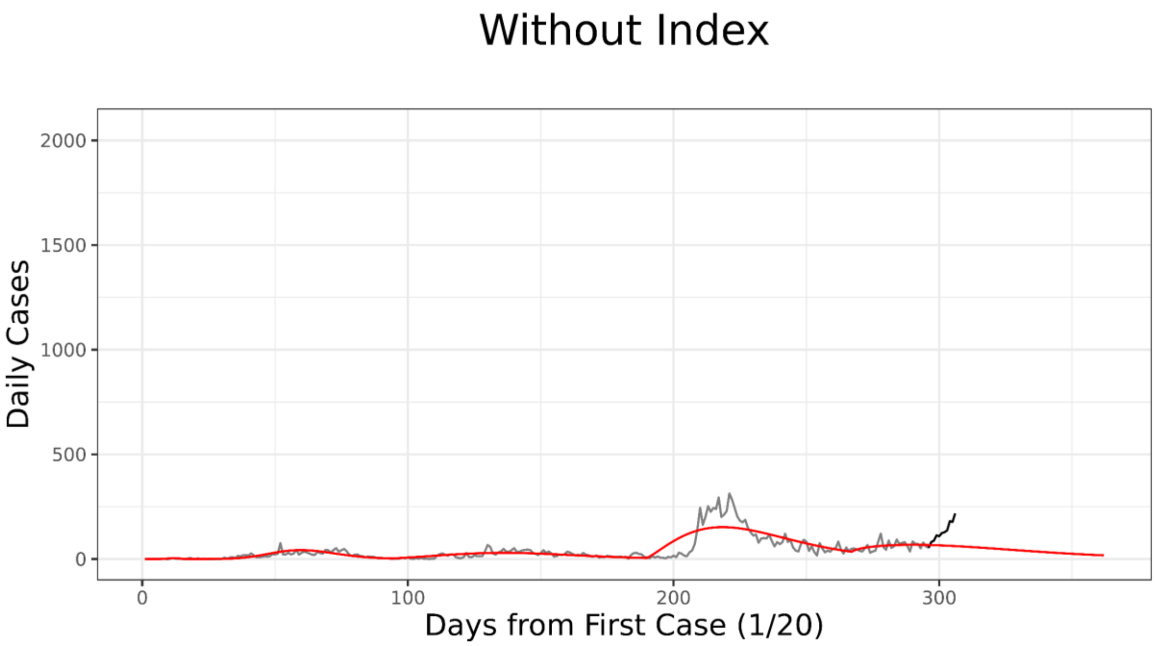

Figure S1: Predicted confirmed cases without any policy implemented

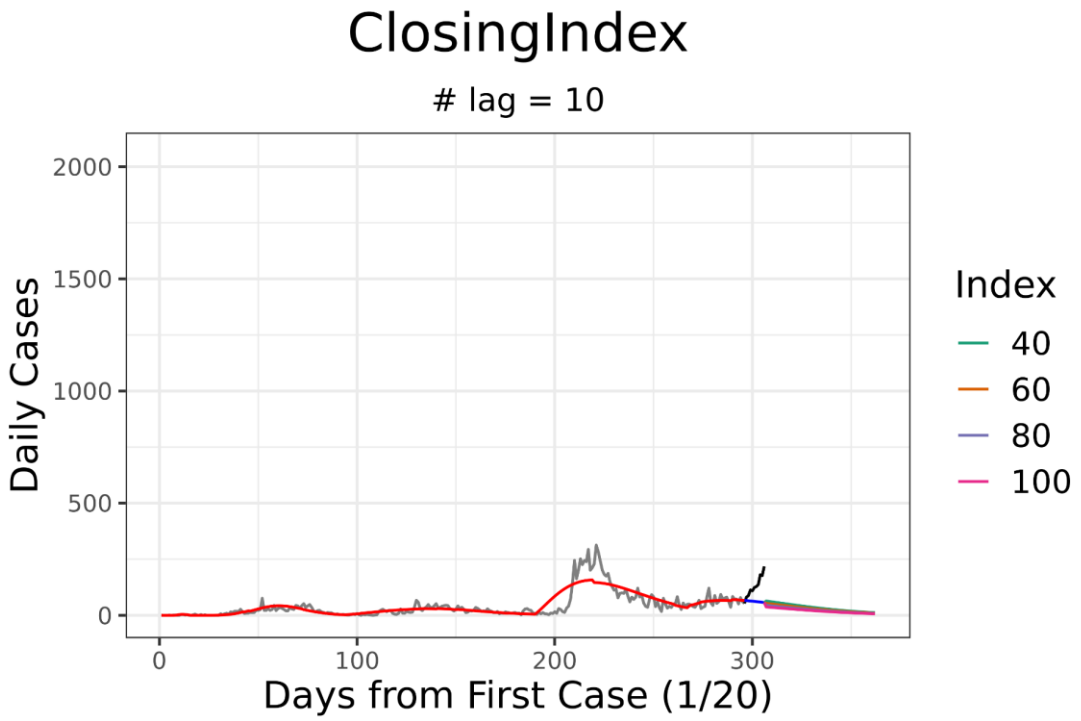

Figure S2: Trends of confirmed cases at different levels of Closing Index with 10-days' lag

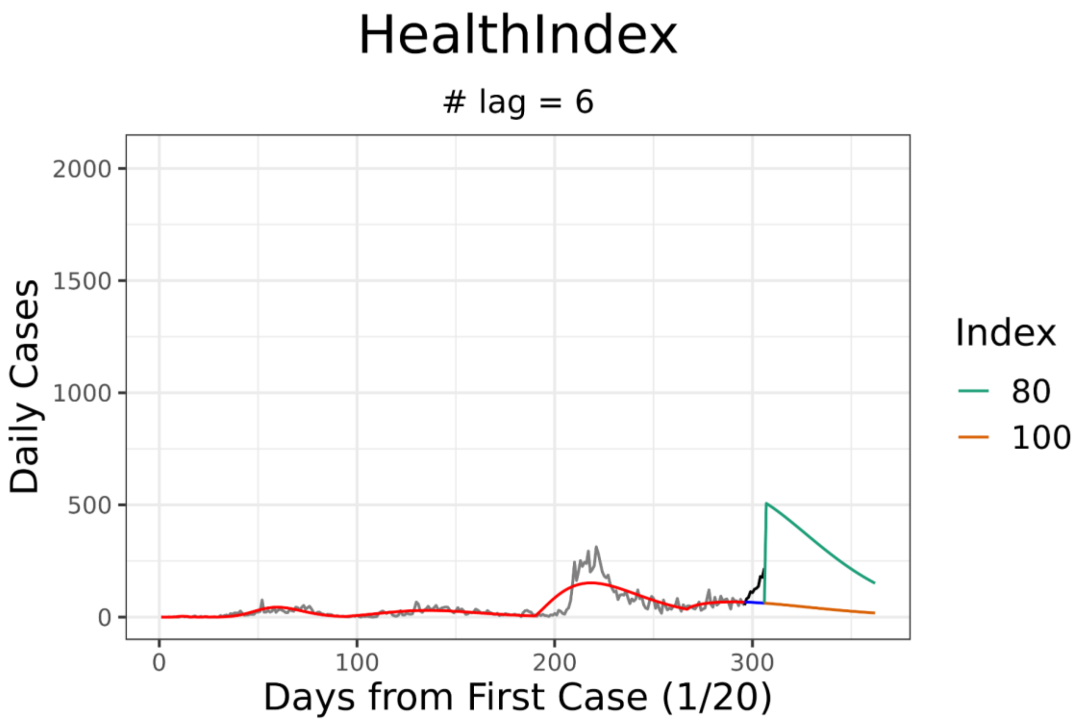

Figure S3: Trends of confirmed cases at different levels of Health Index with 6-days' lag

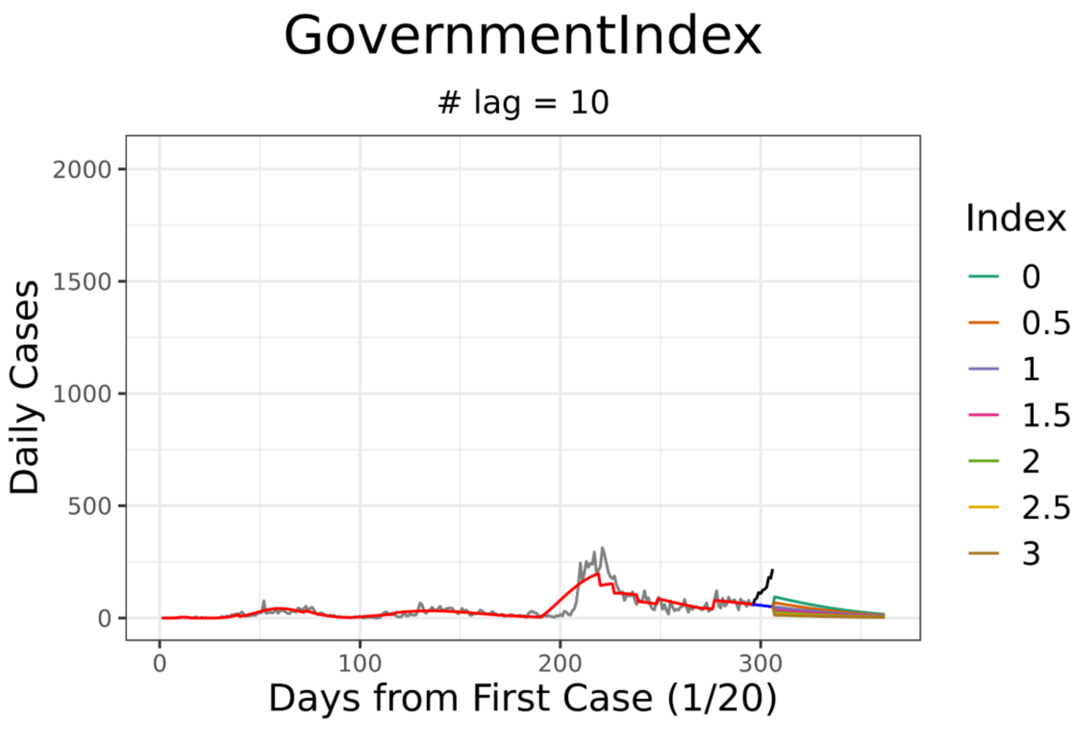

Figure S4: Trends of confirmed cases at different levels of Korean Government Index with 10-days' lag

Non-SeoulMetro Prediction plots

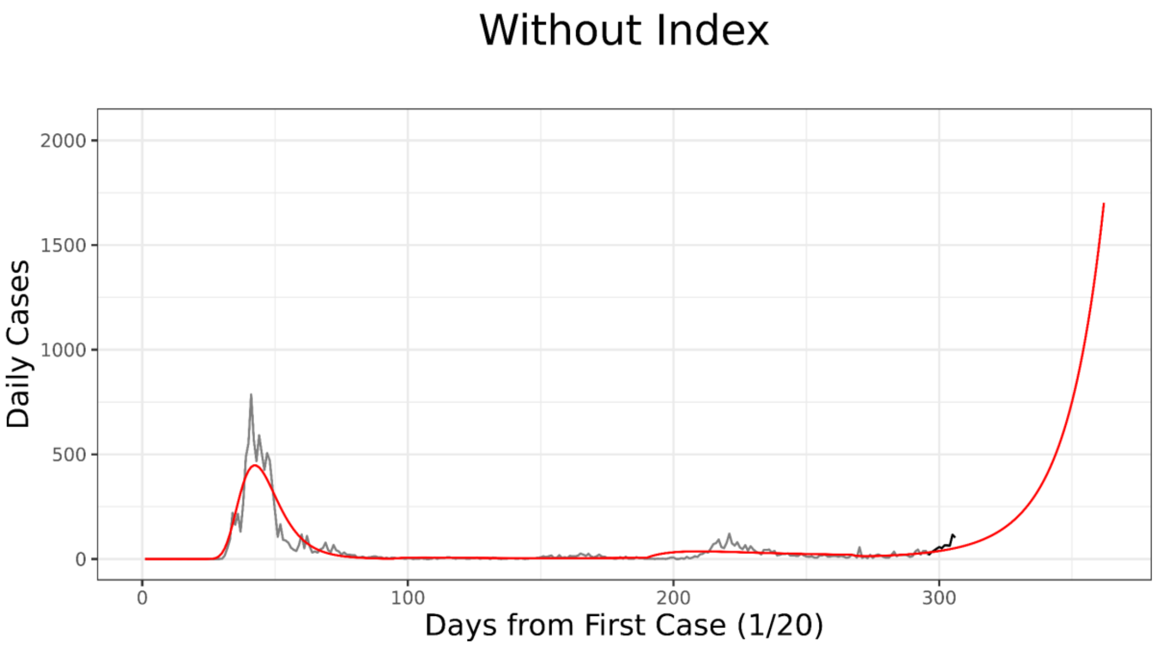

Figure S5: Trend of confirmed cases with no policies implemented

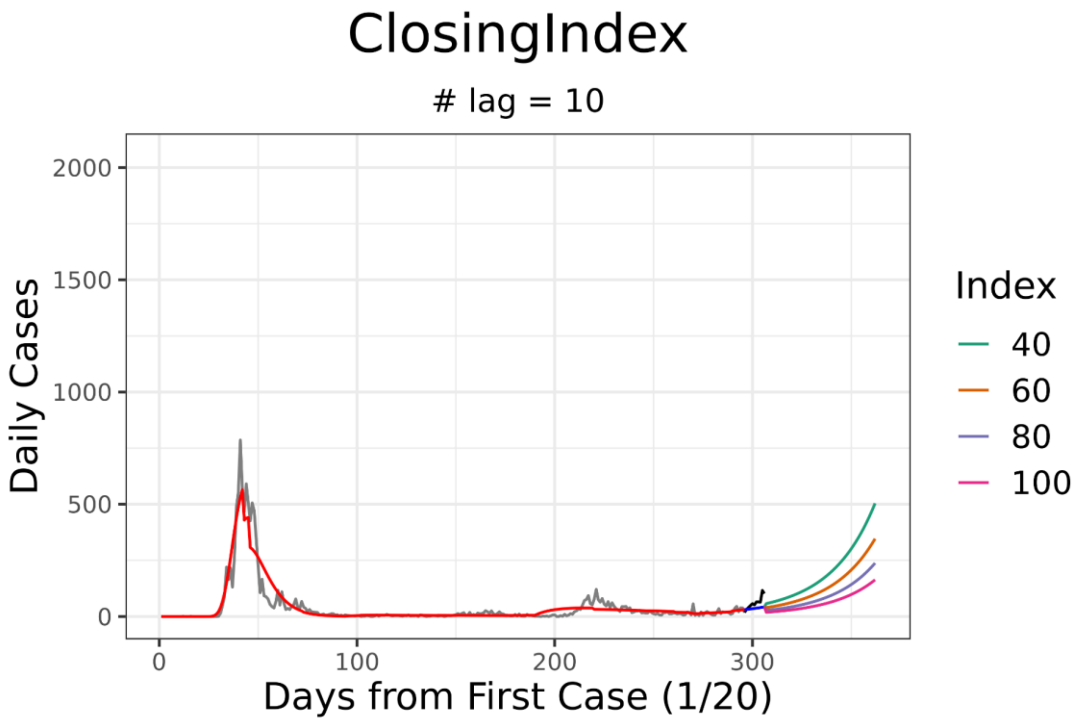

Figure S6: Trends of confirmed cases at different levels of Closing Index with 10-days' lag

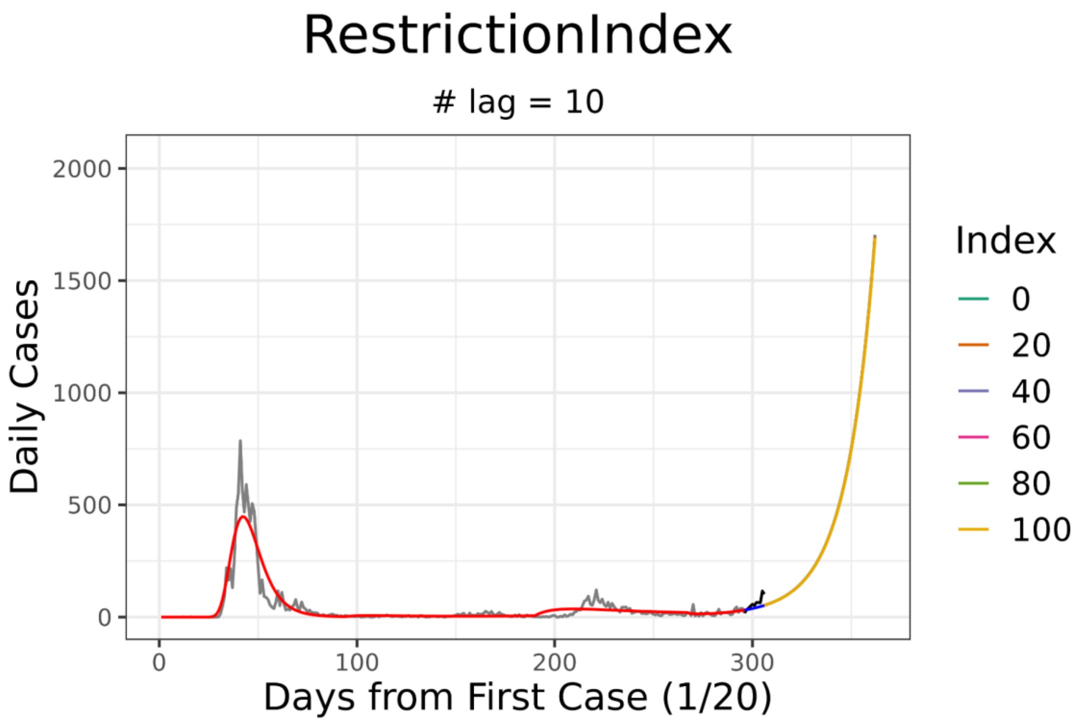

Figure S7: Trends of confirmed cases at different levels of Restriction Index with 10-days' lag

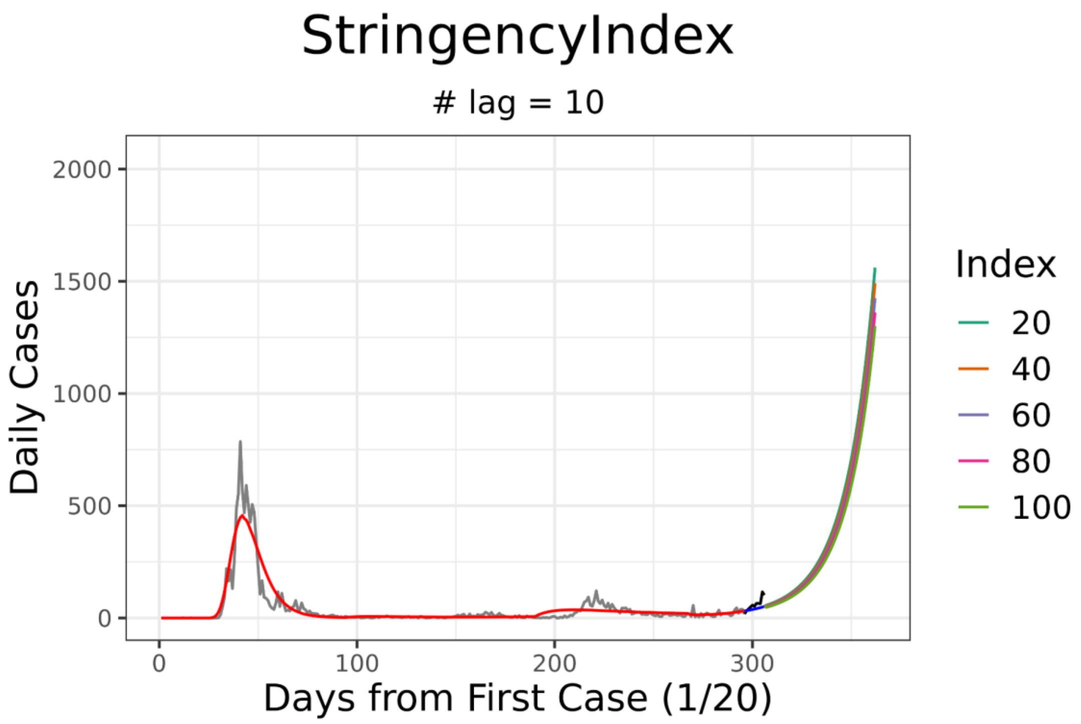

Figure S8: Trends of confirmed cases at different levels of Stringency Index with 10-days' lag

Domestic Prediction Plot

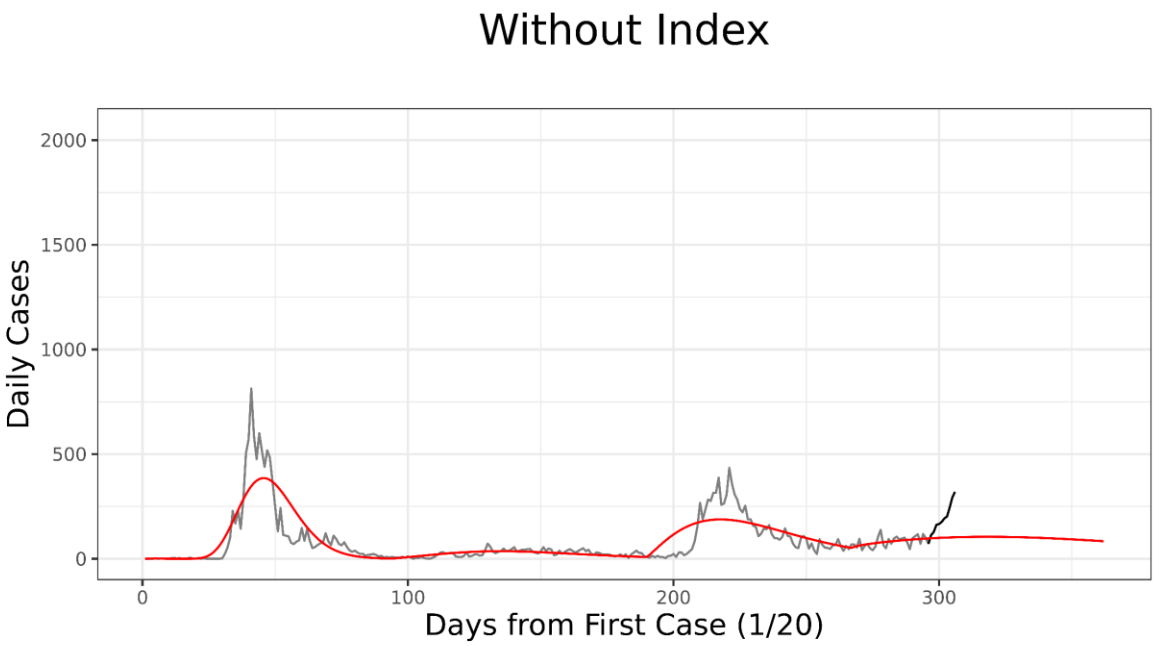

Figure S9: Trends of confirmed cases with no policies implemented

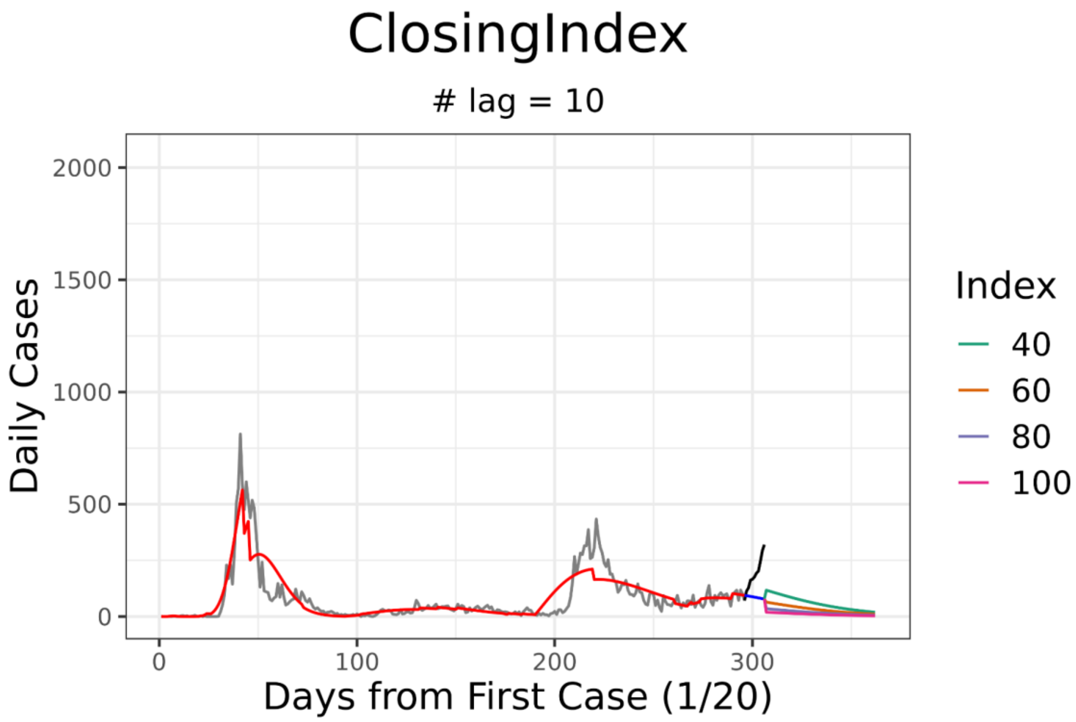

Figure S10: Trends of confirmed cases at different levels of Closing Index

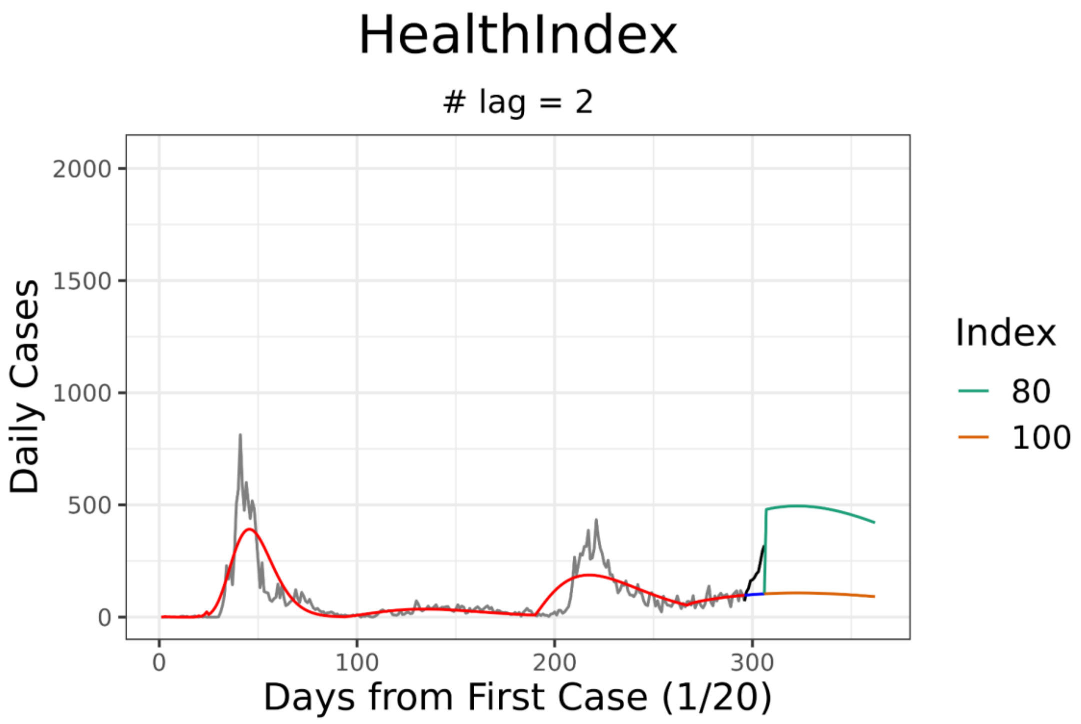

Figure S11: Trends of confirmed cases at different levels of Health Index

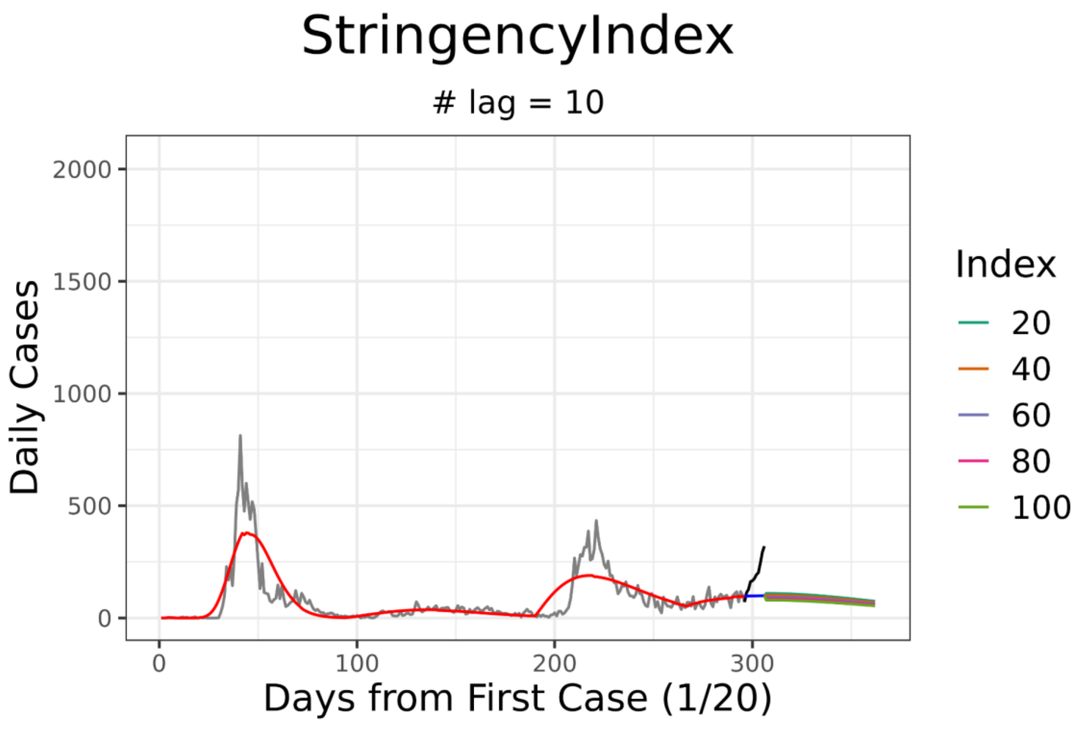

Figure S12: Trends of confirmed cases at different levels of Stringency Index

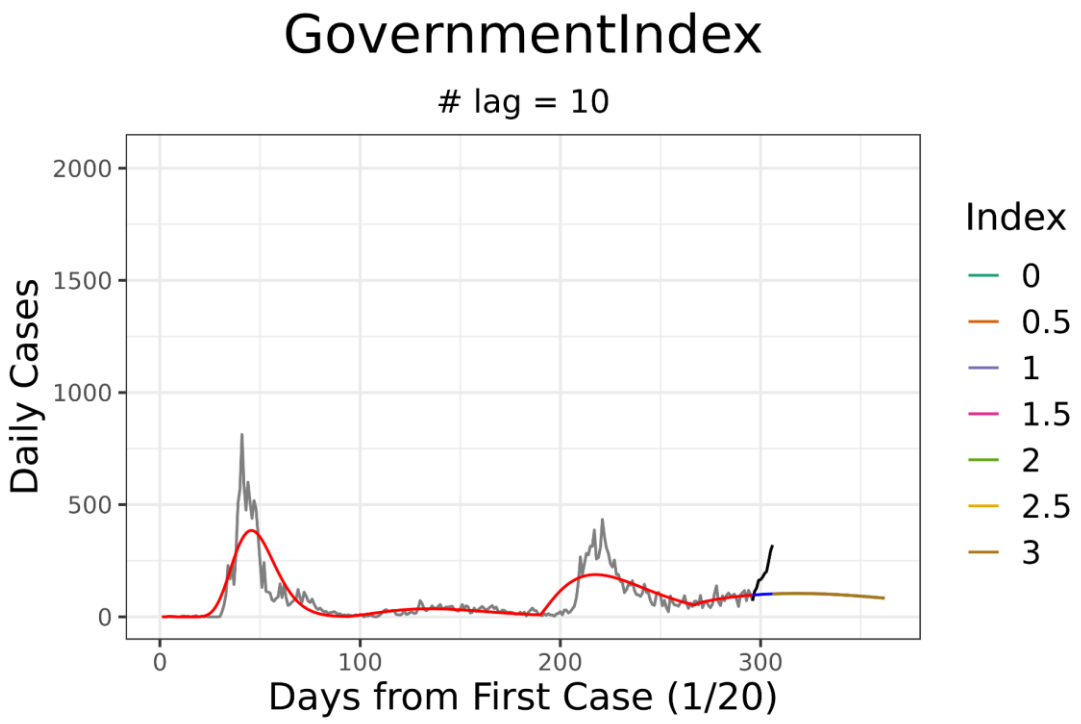

Figure S13: Trends of confirmed cases at different levels of Government Index
